# Proteomic analysis reveals the roles of silicon in mitigating glyphosate-induced toxicity in *Brassica napus* L.

**DOI:** 10.1038/s41598-025-87024-5

**Published:** 2025-01-20

**Authors:** Probir Kumar Mittra, Md Atikur Rahman, Swapan Kumar Roy, Soo-Jeong Kwon, Abhik Mojumdar, Sung Ho Yun, Kun Cho, Seong-Woo Cho, Meiliang Zhou, Tomoyuki Katsube-Tanaka, Sun-Hee Woo

**Affiliations:** 1 Department of Crop Science, Chungbuk National University, Cheongju-si, 28644 Republic of Korea; 2 ABEx Bio-Research Center, East Azampur, Dhaka, 1230 Bangladesh; 3 College of Agricultural Sciences, IUBAT—International University of Business Agriculture and Technology, 4 Embankment Drive Road, Sector 10 Uttara Model Town, Dhaka, 1230 Bangladesh; 4 Digital Omics Research Center, Ochang Center, Korea Basic Science Institute, Cheongju-si, 28119 Republic of Korea; 5 Division of Bio-Analytical Sciences, University of Science and Technology (UST), Daejeon, 34113 Republic of Korea; 6 Department of Agronomy and Medicinal Plant Resources, Gyeongsang National University, 33 Dongjin-Ro, Jinju, 52725 Gyeongnan Korea; 7 Institute of Crop Sciences, Chinese Academy of Agricultural Sciences, 12 South Zhongguancun Street, Haidian, Beijing, 100081 China; 8 Graduate School of Agriculture, Kyoto University, Kitashirakawa Oiwake-cho, Sakyo-ku, Kyoto, 606-8502 Japan

**Keywords:** Key antioxidant proteins, Eco-friendly approach, Herbicide toxicity, Herbicide tolerance, Label-free proteomics, Plant sciences, Environmental sciences

## Abstract

Glyphosate (Gly) is a widely used herbicide for weed control in agriculture, but it can also adversely affect crops by impairing growth, reducing yield, and disrupting nutrient uptake, while inducing toxicity. Therefore, adopting integrated eco-friendly approaches and understanding the mechanisms of glyphosate tolerance in plants is crucial, as these areas remain underexplored. This study provides proteome insights into Si-mediated improvement of Gly-toxicity tolerance in *Brassica napus*. The proteome analysis identified a total of 4,407 proteins, of which 594 were differentially abundant, including 208 up-regulated and 386 down-regulated proteins. These proteins are associated with diverse biological processes in *B. napus*, including energy metabolism, antioxidant activity, signal transduction, photosynthesis, sulfur assimilation, cell wall functions, herbicide tolerance, and plant development. Protein-protein interactome analyses confirmed the involvement of six key proteins, including *L-ascorbate peroxidase*, *superoxide dismutase*, *glutaredoxin-C2*, *peroxidase*, *glutathione peroxidase* (GPX) 2, and *peptide methionine sulfoxide reductase A3* which involved in antioxidant activity, sulfur assimilation, and herbicide tolerance, contributing to the resilience of *B. napus* against Gly toxicity. The proteomics insights into Si-mediated Gly-toxicity mitigation is an eco-friendly approach, and alteration of key molecular processes opens a new perspective of multi-omics-assisted *B. napus* breeding for enhancing herbicide resistant oilseed crop production.

**Supplementary Information:**

The online version contains supplementary material available at 10.1038/s41598-025-87024-5.

## Introduction

In modern agriculture, glyphosate (N-(phosphonomethyl) glycine) is a systemic non-selective herbicide valued for its broad-spectrum effectiveness against a diverse range of weeds. Since its introduction in the 1970s, Gly become an essential component of worldwide agricultural operations, especially with the rise of genetically engineered crops that are resistant to its effect^1^. This compatibility led to more effective weed control, reduced labor costs, and increased agricultural productivity and overall farming efficiency. Despite its widespread use and effectiveness, the indiscriminate application of Gly has raised concerns about its environmental and ecological impacts, particularly on non-target plant species^2^. Scientific research has linked the widespread use of Gly to potential negative impacts, including biodiversity loss, the emergence of Gly-resistant weed species, and disturbance to microbial communities in soil ecosystems^3^.

Understanding the molecular mechanisms underpinning Gly tolerance in plants is critical in developing environmentally suitable approaches to reduce glyphosate-induced toxicity. Silicon (Si) has emerged as a promising agent for enhancing plant resistance against various abiotic and biotic stresses, counteracting the negative impacts of Gly on non-target plants^4,5^. Recent studies have revealed the significance of Si in modulating plant physiological responses, including those related to stress tolerance mechanisms through restoring redox homeostasis^6^. However, the molecular basis of Si-mediated mitigation of Gly toxicity, particularly in *B. napus* L., remain largely unexplored.

*Brassica napus* L., also commonly known as rapeseed or canola, is an economically significant oilseed crop that substantially contributes to the edible oil sector. It contains high levels of essential fatty acids, carbohydrates, proteins, vitamins, and minerals and plays an important role in the human diet by providing a variety of nutritional advantages^7,8^. It also provides a sustainable source for biodiesel and biolubricant production. However, it has been observed to exhibit lower yield and modified physiological processes when exposed to Gly, highlighting the need for sustainable farming approaches that minimize these harmful effects^9^. Due to the distinct genetic makeup and the biochemical compositions, this plant is an ideal model for advanced study on the resilience mechanisms, especially when it comes to environmental stresses such as glyphosate-induced toxicity^10,11^. Hence, this work utilizes the resilient features of *B. napus* to uncover the underlying proteomic responses and adaptive mechanisms used by this plant to counteract the Gly-induced stress, integrating the physiological observations with state-of-the-art proteomic techniques.

The advent of proteomics has provided insights into the complex molecular dynamics of plant responses to various environmental stressors^12^. Label-free quantification is a widely used proteomic technique that enables comprehensive and unbiased assessment of protein expression changes without requiring complex labeling processes^13^. This contrasts with traditional gel-based approaches, which, while effective, are sometimes constrained by their lower throughput and sensitivity. Researchers may now unravel the intricate networks of protein interactions as well as modifications that help plants sustain herbicide-induced stress by exploiting the capabilities of label-free proteomics^14^.

**Fig. 1:**
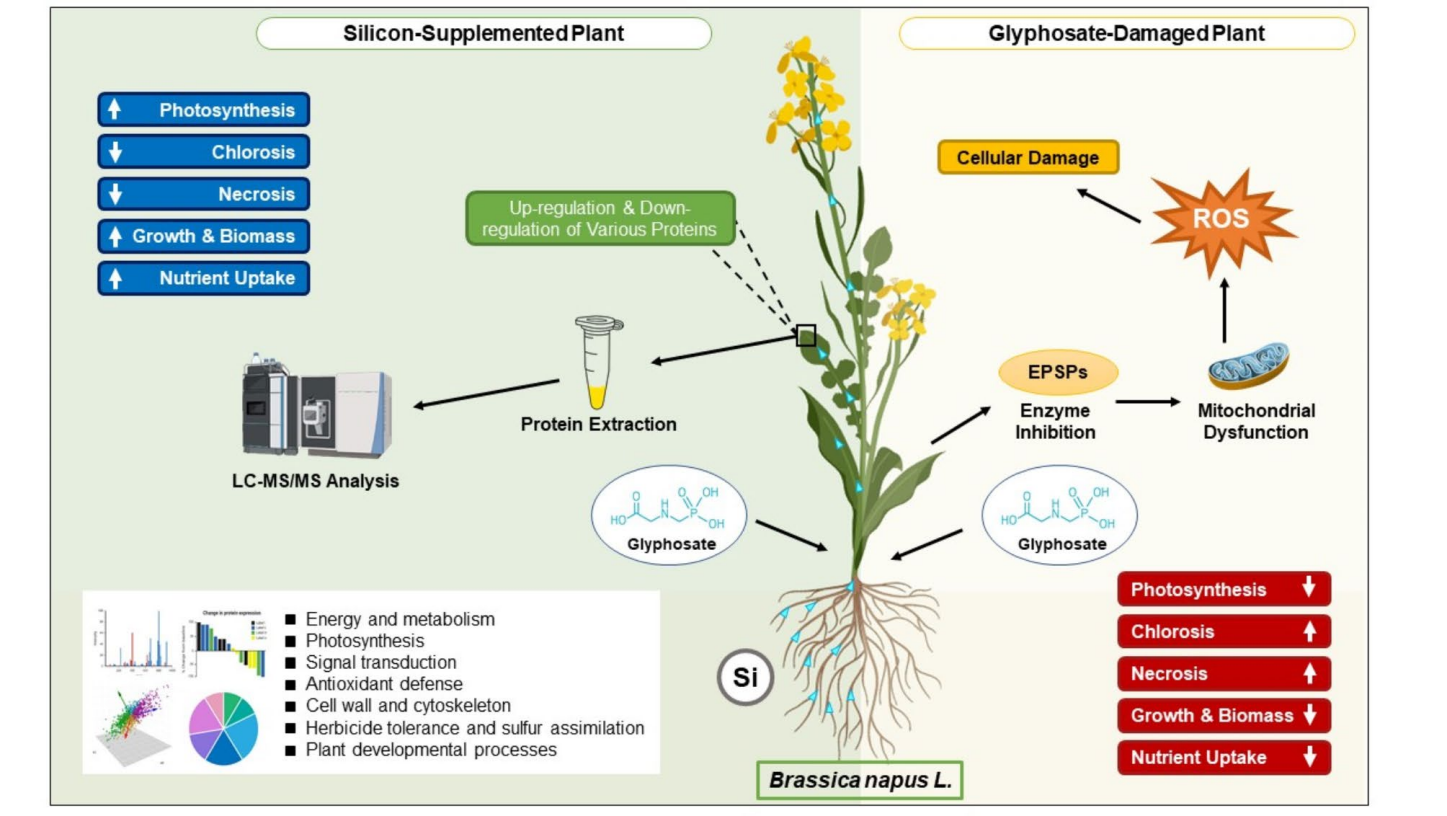
Graphical abstract of Si-mediated Gly toxicity tolerance in *Brassica napus*. Glyphosate application inhibits the enzyme 5-enolpyruvylshikimate-3-phosphate synthase (EPSPs), triggering mitochondrial dysfunction, the overproduction of reactive oxygen species (ROS), and consequent cellular damage manifested as chlorosis, necrosis and impaired growth. In contrast, Si application under Gly stress boosts photosynthesis, biomass accumulation, growth, and nutrient uptake while alleviating chlorosis and necrosis. Proteomic analysis reveals that Si treatment activates some diverse protective mechanisms, including energy and metabolism, photosynthesis, signal transduction, antioxidant defense, cell wall and cytoskeleton, herbicide tolerance and sulfur assimilation, and plant developmental processes. Among these mechanisms, several key proteins related to antioxidant activity, sulfur assimilation, and herbicide tolerance contribute to glyphosate tolerance as confirmed by interactome analysis in the main text. The proteomic alterations enhance Gly-tolerance in *Brassica napus* under Si supplementation. Abbreviation, EPSPs, 5-enolpyruvylshikimate-3-phosphate synthase; ROS, reactive oxygen species; Si; silicon; Gly, glyphosate.

Thus, our study aims to elucidate the protective implications of Si in *B. napus* exposed to Gly, using a label-free proteomic approach to map the protein expression landscape. We sought to understand the molecular mechanisms behind silicon’s mitigating effects by studying the differential expression of proteins involved in critical biological functions such as energy metabolism, photosynthesis, signal transduction, and antioxidant defense. Our findings reveal a considerable alteration in the proteome of *B. napus* leaves, as evidenced by the differential expression of key proteins involved in antioxidant responses, sulfur absorption, and herbicide tolerance. These insights not only improve our understanding of the molecular responses of *B. napus* to Gly treatment but also open the path towards developing targeted breeding and management techniques to enhance Gly tolerance in crops (Fig. 1).

## Materials and methods

### Plant culture and treatments

The healthy seeds of *Brassica napus* were obtained from the National Institute of Crop Research (NICR), Rural Development Administration (RDA), Muan, South Korea as a gift. The *B. napus* seeds were subjected to surface sterilization using a 1% sodium hypochlorite solution for 20 min, followed by thorough washing with sterile purified water three times. Subsequently, the seeds were transferred to the germination tray and placed in a controlled environment for five days, with a photoperiod of 14 h of light and 10 h of darkness, at a temperature of 25 °C. A growth chamber was used to maintain a relative humidity of 65%, and 14 h light with a light density of 150 molm^− 2^s^− 1^, 10 h darkness, with 25 °C temperature. After germination, healthy seedlings were transplanted into plastic boxes containing nutrient solutions. The seedlings were hydroponically cultivated by using the Hoagland nutrient solution^15^. The solution was changed every 2 days to prevent potential precipitation of nutrients, Gly and Si. The air was supplied continuously through the electric air pump in the nutrient solution for sufficient oxygen. After 21 days of cultivation, the seedlings underwent exposure to different nutrient solutions, either with or without Gly and Si: Control; Gly (40 µM); Gly (40 µM) + Si (0.5 mM), Si (0.5 mM). The experiments included three biological replicates and five technical replicates for each treatment. The plants were harvested after seven days of the treatment application. The samples were immediately frozen in liquid nitrogen and stored at -80 °C until they were ready for molecular and biochemical analyses.

### Measurement of morphological features

The shoot and root heights of the plants were measured (cm), and their fresh weights were measured with electronic balance immediately (EPG214C, Pine Brook, USA). The samples were dried and dry weights were then recorded.

## Analysis of chlorophyll contents

The photosynthetic pigments concentration was determined by following a slightly modified method^16^. In brief, 0.04 g of plant material was homogenized with dimethyl sulfoxide (DMSO). The homogenate was incubated at 65 °C for 4 h, then centrifuged at 10,000 rpm for 10 min. The supernatant’s absorbance was measured at 452, 644 and 663 nm. The photosynthetic pigments concentrations were calculated using the following equations^17^:

$$Chlorophyll\;a\left( {\mu g/g} \right) = 10.3 \times A663 - 0.918 \times A644$$

$$Chlorophyll\;b\left( {\mu g/g} \right) = 19.7 \times A644 - 3.878 \times A644$$

$$\:Total\:chlorophyll=Chlorophyll\:a+Chlorophyll\:b$$

$$\:Carotenoids\:\left(\mu\:g/g\right)=4.2\:\times\:A452\:\times\:(0.0264\:\times\:Chlorophyll\:a+0.426\:\times\:Chlorophyll\:b$$

### Measurement of ROS levels (H2O2 and O2•−)

The concentration of hydrogen peroxide (H_2_O_2_) was determined according to previously described method^18^. Superoxide (O_2_^•−^) levels were determined using the previously described extinction coefficient method^19^.

### Analysis of antioxidant enzymes (SOD, CAT, APX and GST)

Leaf tissue was homogenized individually using a mortar and pestle with 100 mM potassium phosphate buffer (pH 7.0). The homogenate was centrifuged at 8000 rpm for 10 min, and the supernatant was collected for enzyme activity analysis. SOD activity was measured by adding 100 µL of plant extract to a mixture containing 50 mM NaHCO_3_ (pH 9.8), 0.1 mM EDTA, and 0.6 mM epinephrine, following the previously described protocol^20^. After four min, the formation of adrenochrome was recorded at 475 nm. To determine CAT activity, a mixture of 100 µL of plant extract and 6% H_2_O_2_, 100 mM potassium phosphate buffer (pH 7.0) was used. The absorption of the solution was measured at 240 nm at 30-second intervals, using an extinction coefficient of 0.036 mM⁻¹cm⁻¹. APX activity was assessed by mixing 0.1 mL of plant extract with 0.1 mM EDTA, 0.1 mM H_2_O_2_, 50 mM potassium phosphate buffer (pH 7.0), and 0.5 mM ascorbic acid with, according to the previously described method^21^. The absorbance of the mixture was measured at 290 nm, with APX activity calculated using an extinction coefficient of 2.8 mM⁻¹cm⁻¹. GST activity was determined by adding 100 µL of the sample extract in 1.5 mM GSH, 1 mM 1-chloro-2,4-dinitrobenzene (CDNB) and 100 mM Tris-HCl buffer (pH 6.5), following the previously described method^22^. The absorbance of the mixture was measured at 340 nm.

### Extraction and measurement of proteins

Protein extraction from the tissue was conducted using a TCA/acetone-based previously described method^23^. Leaf samples were ground with liquid nitrogen using a mortar and pestle. A 0.5 g sample was homogenized in a solution of 10% TCA, 0.07% (v/v) 2-mercaptoethanol and ice-cold acetone. The mixture was sonicated for 10 min, incubated at -20 °C for 1 h., and centrifuged at 9000 g for 20 min at 4 °C. The protein pellet was washed with cold acetone, dried and reconstituted in a buffer. After 1 h at room temperature, the mixture was centrifuged at 20,000 g for 20 min at 25 °C, and the protein concentration was measured using the Bradford assay^24^.

### Purification and digestion

The leaf proteins of *B. napus* were purified using the methanol-chloroform method^25^. Initially, 450 µL of water with 150 µL of chloroform were added, vortexed and centrifuged at 20,000 g for 10 min. After removing the aqueous phase, the organic phase was mixed with 450 µL of methanol. After another centrifugation under the same conditions, the supernatant was discarded. The resulting pellet was air-dried for 10 min and re-dissolved in 50 mM NH_4_CO_3_. Each sample was reduced by incubating with 50 mM DTT at 56 °C for 30 min. IAA was then added, and the samples were kept at 37 °C temperature for 30 min. Trypsin was added to the sample and incubated for 16 h, following previously described method^26^. To prepare the trypsin solution, 200 µL of Trypsin Resuspension Buffer (PROMEGA) was added to a vial containing 20 µg of sequencing-grade modified trypsin (porcine). The samples were purified using previously described method^23^, transferred to new tubes and prepared for MS analysis.

### LC MS analysis

The extracted peptides were analyzed using a mass spectrometer (Thermo Fisher LTQ Orbitrap, Germany) combined with an Agilent 1100 nano-flow HPLC system with a standard ion source. The setup involved two columns. A three-way tee connector was used to connect the pre-column, waste line, and analytic column (C18 AQ, 3 m, 100 m x 15 cm, Nano LC, USA). A 10 µL volume of peptide solution was loaded onto the trap column (75 μm x 2 cm, nano Viper C18, 3 μm, Thermo Fisher Scientific). Two distinct solvent phases, A and B, were utilized, consisting of 0.1% formic acid (FA) in water (solvent A) and 0.1% FA in 100% ACN (solvent B). The peptides were desalted and concentrated on the trap column for 10 min using solvent (A) A fixed linear gradient over 150 min was used to elute the peptides with solvent B at a flow rate of 300 nL/min. To ensure cleanliness, the column was washed with at least ten column volumes of 100% solvent (B) Peak validity was confirmed using a standard peak shape method, considering only those peaks with well-defined symmetrical shapes as valid^27^. In full MS scans, the top five peaks were fragmented using data-dependent acquisition with 35% normalized collision energy. Peptide ions were introduced at 2.2 kV capillary voltage. The MS settings included a 0.5 Da mass exclusion width, 180 s dynamic exclusion, and spectra were recorded from 50 to 2000 m/z.

### Validation of peptide

The MS/MS spectra were analyzed using Mascot Daemon, against the GPR database (http://www.uniprot.org.). Search parameters included a ± 1.5 Da precursor mass tolerance, ± 0.8 Da fragment mass tolerance, up to two missed trypsin cleavages, and carbamidomethyl cysteine modification. Mascot’s ion score threshold was set at 0.05, and peptide identification was validated with a 1% FDR (false discovery rate) threshold. The peptide score, calculated as -10 Log, this peptide needed a homology score with *P* < 0.01. Label-free quantification followed the previously described protocol^28^, excluding reverse decoy matches and impurities. Proteins required identification with at least two distinct peptides and quantification in at least two technical replicates across four biological replicates, with average intensities calculated for each group.

### Statistical analysis

Statistical analysis used a two-sided t-test, with FDR (false discovery rate) correction for multiple comparisons, conducted in Perseus statistical software with default settings. Data were normalized by linear regression and transferred to excel for detailed analysis. Peptides matching common impurities were filtered out and at least three biological replicates were used for relative quantification and protein identification.

### Analysis of bioinformatics

Gene-encoded proteins were analyzed for their cellular components, molecular functions, and biological processes using the DAVID Bioinformatics (https://david.ncifcrf.gov/). This DAVID tool provides a comprehensive platform for the analysis of gene lists and functional annotation, integrating various genomic resources to elucidate gene functions and their biological significance. The abundance pattern of proteins were visualized with a heatmap generated by ClustVis (https://biit.cs.ut.ee/clustvis/). Fold change in protein levels between the control and the treatments was calculated by comparing the average normalized values, with *p*-value analysis determining significance. Upregulation and downregulation patterns were analyzed using the previously described methodology^29^, with a significance threshold of ≤ 0.05. Furthermore, the KEGG (http://www.genome.jp/kegg/pathway.html) was used to confirm the involvement of these proteins in plant molecular processes^30^.

### Interactome analyses

Protein-protein interactions were examined with STRING database (https://string-db.org/), which classified the interactions according to the primary functions of the candidate proteins. The resulting network was visualized by Cytoscape (https://cytoscape.org/)^31^. The 3D structures of key proteins related to sulfur assimilation, antioxidant activity, and Gly tolerance were modeled using the SWISS-MODEL (https://swissmodel.expasy.org/) web tool. Models were chosen based on the Global Model Quality Estimation (GMQE) score, which ranges from 0 to 1, with higher scores indicating greater confidence and accuracy in the protein models.

### Data analyses

Data analysis was conducted using ANOVA, and the results are displayed as the mean of three biological replicates with standard error (SE). Comparisons between treatments were performed using Student’s *t*-test, considering a *p*-value of ≤ 0.05 as statistically significant.

Graphical presentations were generated using GraphPad Prism software (Version 9.0).

## Results

### Morphological features

**Fig. 2:**
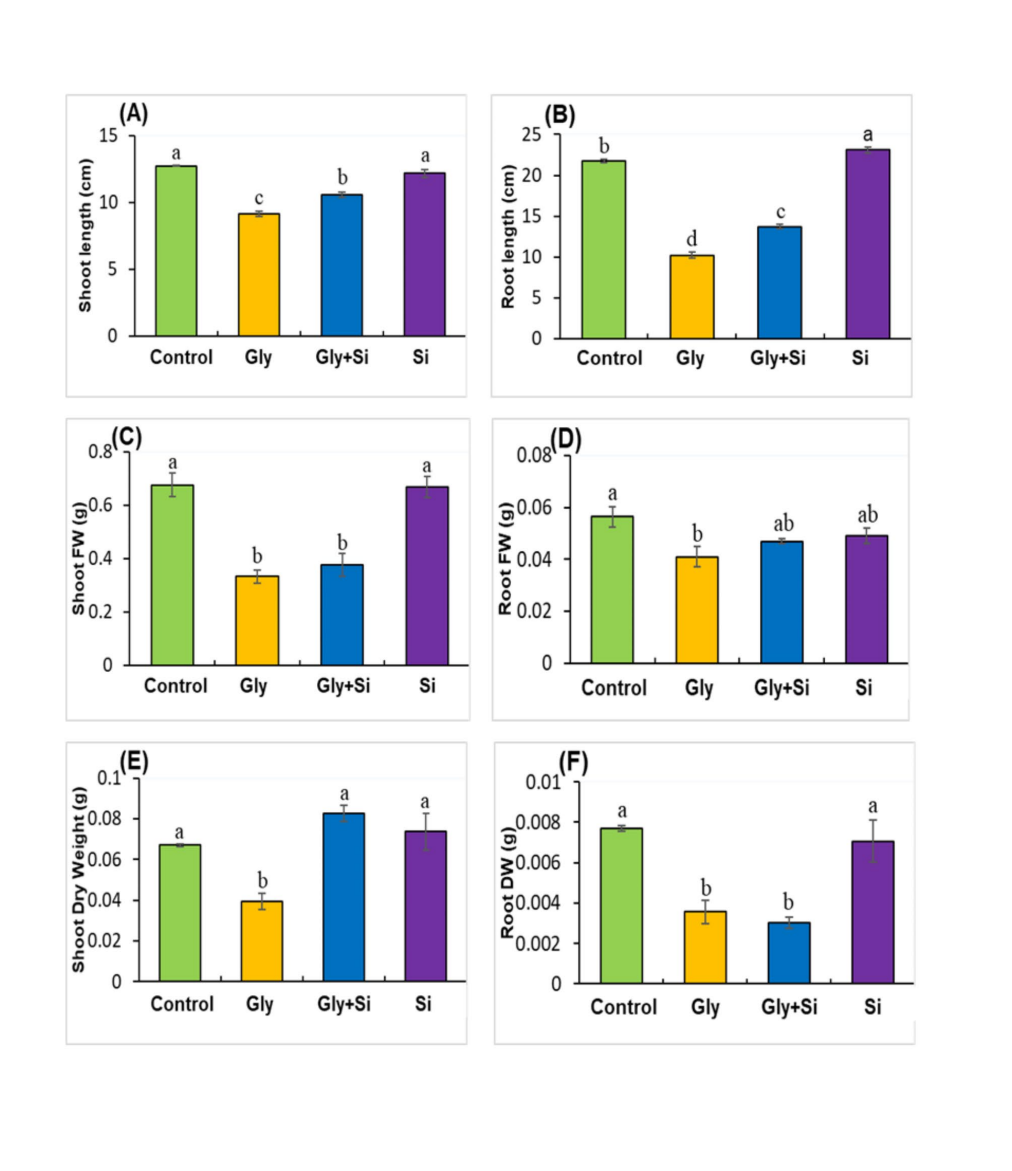
Effect of exogenous Gly and Si on shoot length (**A**), Root length (**B**), shoot fresh weight (**C**), root fresh weight (**D**), shoot dry weight (**E**) and root dry weight (**F**) in *Brassica napus* seedlings with 40 µM Gly and 0.5 mM Si. Abbreviation, Gly, glyphosate, Si, silicon. Each value represents the mean of three replicates ± SE. Different letters indicate significant differences at *p* < 0.05 among treatments by Tukey’s test.

Gly toxicity significantly inhibited the growth of *B. napus*, causing notable reductions in root and shoot lengths, as well as a substantial decrease in overall plant biomass. However, the addition of Si restored these characteristics (Fig. 2). Specifically, Gly toxicity reduced shoot length by 28.27% and root length by 53.13% compared to control plants. Similarly, shoot fresh weight, root fresh weight, shoot dry weight, and root dry weight decreased by 53.63%, 41.29%, 27.21%, and 50.76%, respectively (Fig. 2).

### Chlorophyll contents

**Fig. 3:**
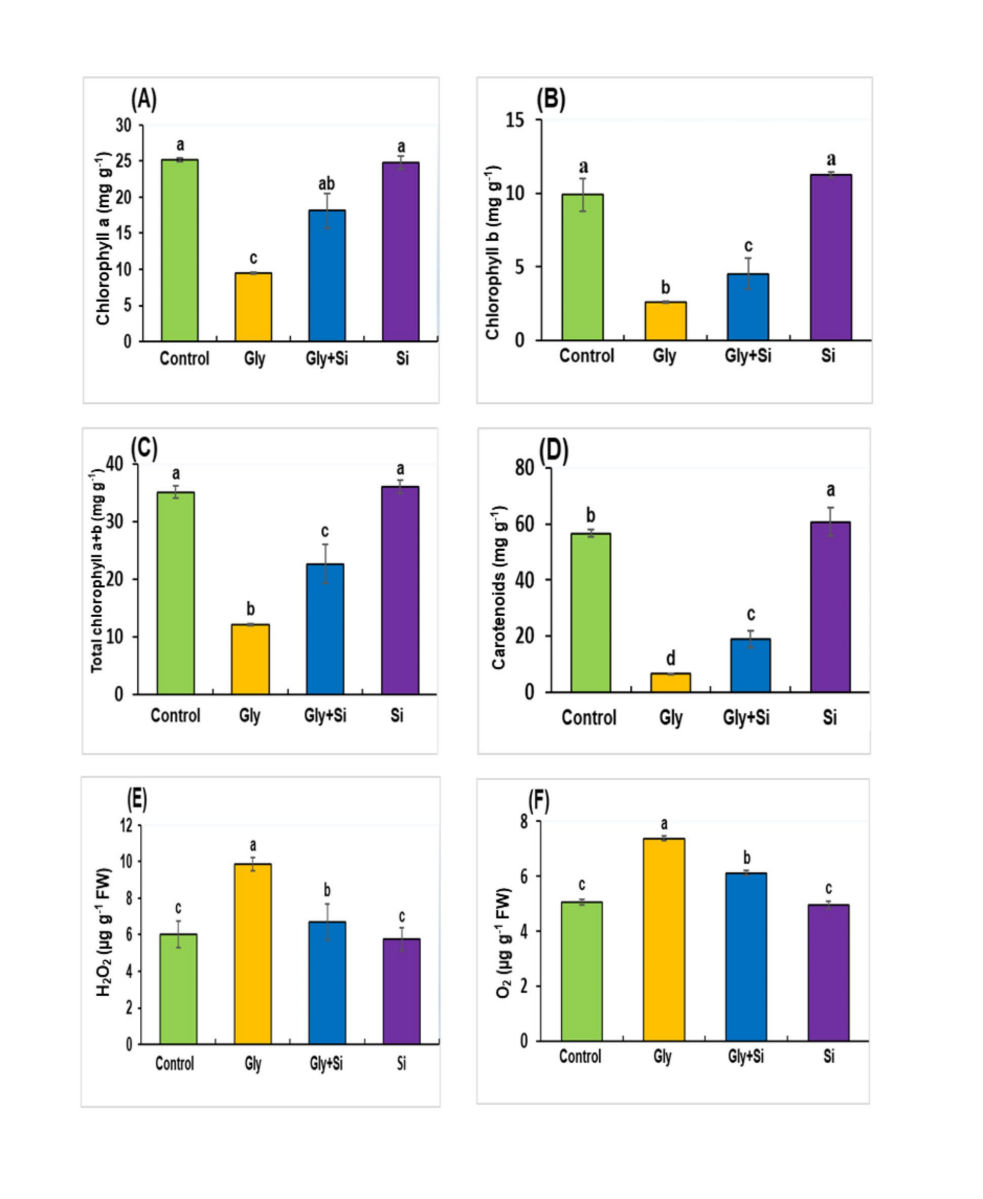
Effect of exogenous Gly and Si on chlorophyll a (**A**), chlorophyll b (**B**), total chlorophyll a + b (**C**), carotenoids (**D**), H_2_O_2_ content (**E**), O_2_•^−^ content (**F**) in *Brassica napus* leaves. The normal growth condition (absence of Gly and Si; control), Gly (40µM), Gly + Si (40µM Gly + 0.5 mM Si), and Si (0.5 mM Si). Abbreviation, Gly, glyphosate; Si, silicon. Each value represents the mean of three replicates ± SE. Different letters indicate significant differences at *p* < 0.05 among treatments by Tukey’s test.

Due to Gly toxicity, photosynthetic pigments, including chlorophyll a, chlorophyll b, total chlorophyll and carotenoids were drastically reduced by 62.30%, 73.56%, 65.47% and 88.50% respectively compared to seedlings under control treatments (Fig. 3A-D). Alternatively, the addition of Si to Gly (40 µM) treatment significantly restored these pigments in *B. napus*. Specifically, Chlorophyll a, Chlorophyll b, total Chlorophyll and carotenoids increased by 90.68%, 73.54%, 86.97% and 191.76%, respectively, compared to Gly-treated seedlings (Fig. 3A-D).

### Changes of H2O2 and O2•-

Under Gly stress, the H_2_O_2_ content in *B. napus* increased by 63.82% compared to control plants (Fig. 3E). Exogenous supplementation of Si significantly reduced H_2_O_2_ and O_2_^•-^. Additionally, the concentration of O_2_^•-^ raised by 45.67% under Gly-treated plants compared to the plants under control treatments (Fig. 3F). However, the addition of Si considerably decreased O_2_•^-^, indicating that Si effectively mitigates the overproduction of O_2_•^-^ under Gly stress. No significant difference was detected between the control plants and those treated exclusively with Si. These findings suggest that Si was active in response to Gly-toxicity that significantly mitigated oxidative stress in *B. napus*.

### Changes in antioxidant enzyme activity

**Fig. 4:**
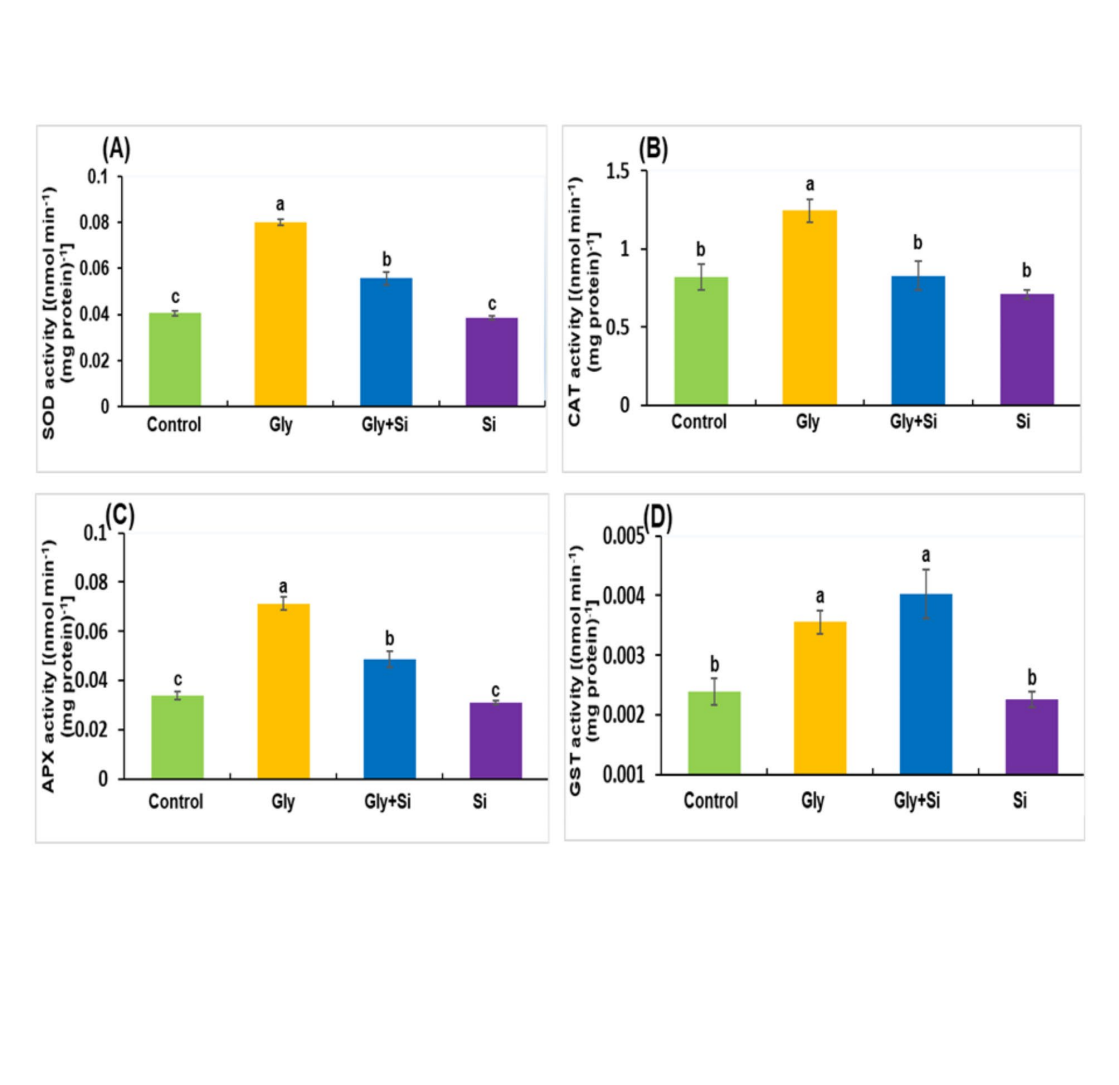
Effect of exogenous Gly and Si on enzyme activities SOD (**A**), CAT (**B**), APX (**C**), GST (**D**) in *Brassica napus* leaves. Each value represents the mean of three replicates ± SE. Different letters indicate significant differences at *p* < 0.05 among treatments by Tukey’s test.

Compared to the control plants, Gly treatment resulted in a prominent elevation in SOD activity (Fig. 4A). Si supplementation remarkably reduced this SOD activity, whether or not Gly was present (Fig. 4A). Under Gly treatment, CAT and APX activities significantly increased compared to control plants (Fig. 4B, C). However, these activities decreased following Si supplementation, both with and without Gly treatment (Fig. 4B, C). Additionally, GST activity consistently increased under Gly treatment compared to the control (Fig. 4D). Notably, the addition of Si to Gly treatment further enhanced GST activity, whereas Si treatment alone resulted in a reduction in GST activity (Fig. 4D).

### Gly-induced alterations of ***B. napus*** proteome

**Fig. 5:**
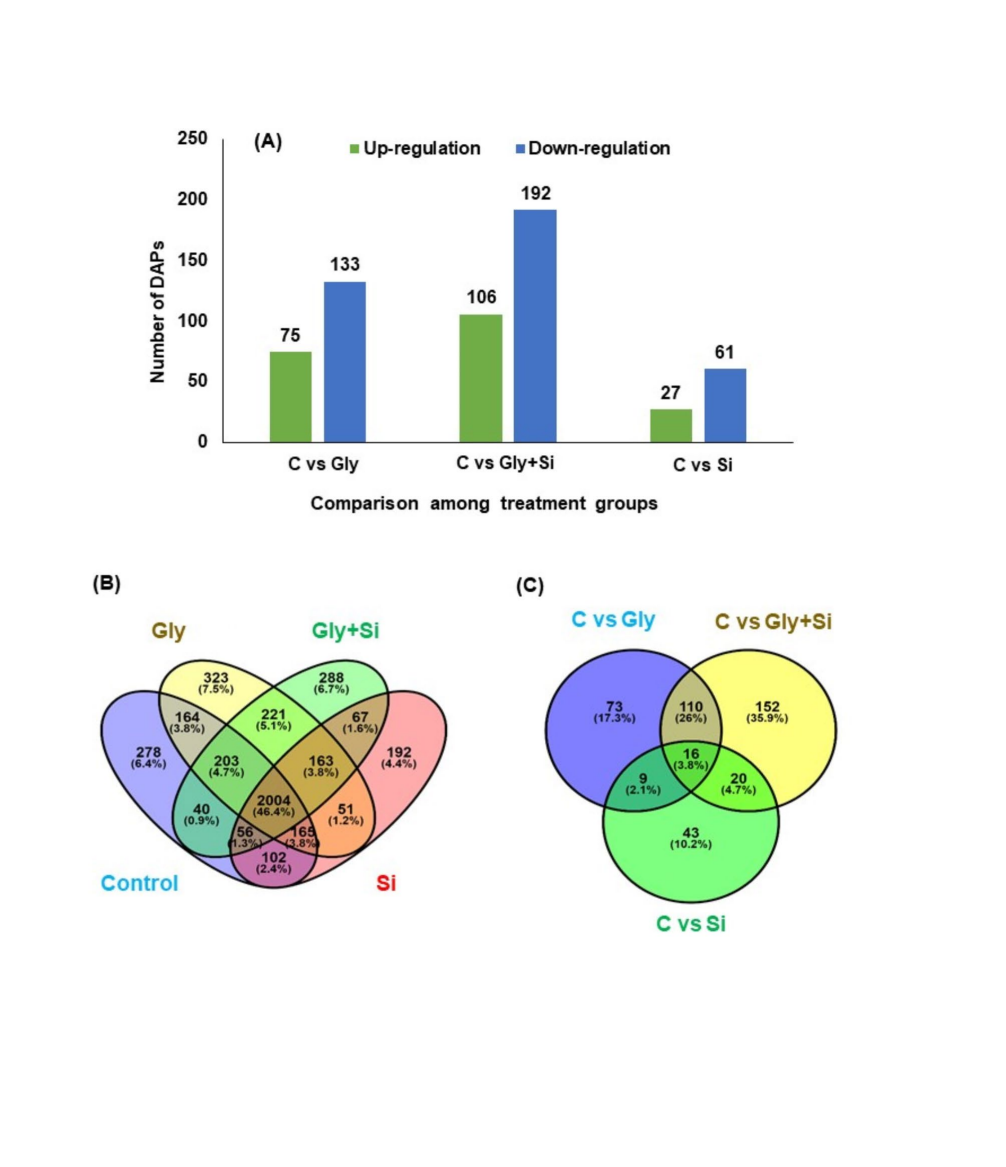
Identification and statistics analysis of identified proteins under different treatment groups. (**A**) number of up or downregulated proteins between the control group and different treatments, (**B**) venn diagram analysis for common proteins (CP), and (**C**) venn diagram analysis for upregulated and downregulated proteins. Abbreviation, C, control; Gly, glyphosate; Si, silicon.

In *B. napus*, Gly-toxicity substantially altered the responses of the global leaf proteome. A proteomic approach was used to identify a total of 4407 proteins, from which many key functional proteins were screened. A proteomic approach identified a total of 4,407 proteins, among which several key functional proteins were screened. Across all treatment groups, 594 differentially abundant proteins (DAPs) were identified (Fig. 5A, Suppli. Table S3). Comparison between the control plants and the Gly-treated plants (C vs. Gly), we identified 208 DAPs, with 75 proteins showing increased abundance and 133 showing decreased abundance. In the C vs. Gly + Si comparison, 106 and 192 proteins were differentially abundant, respectively, while in the C vs. Si comparison, 27 and 61 proteins were differentially abundant (Fig. 5A).

**Fig. 6:**
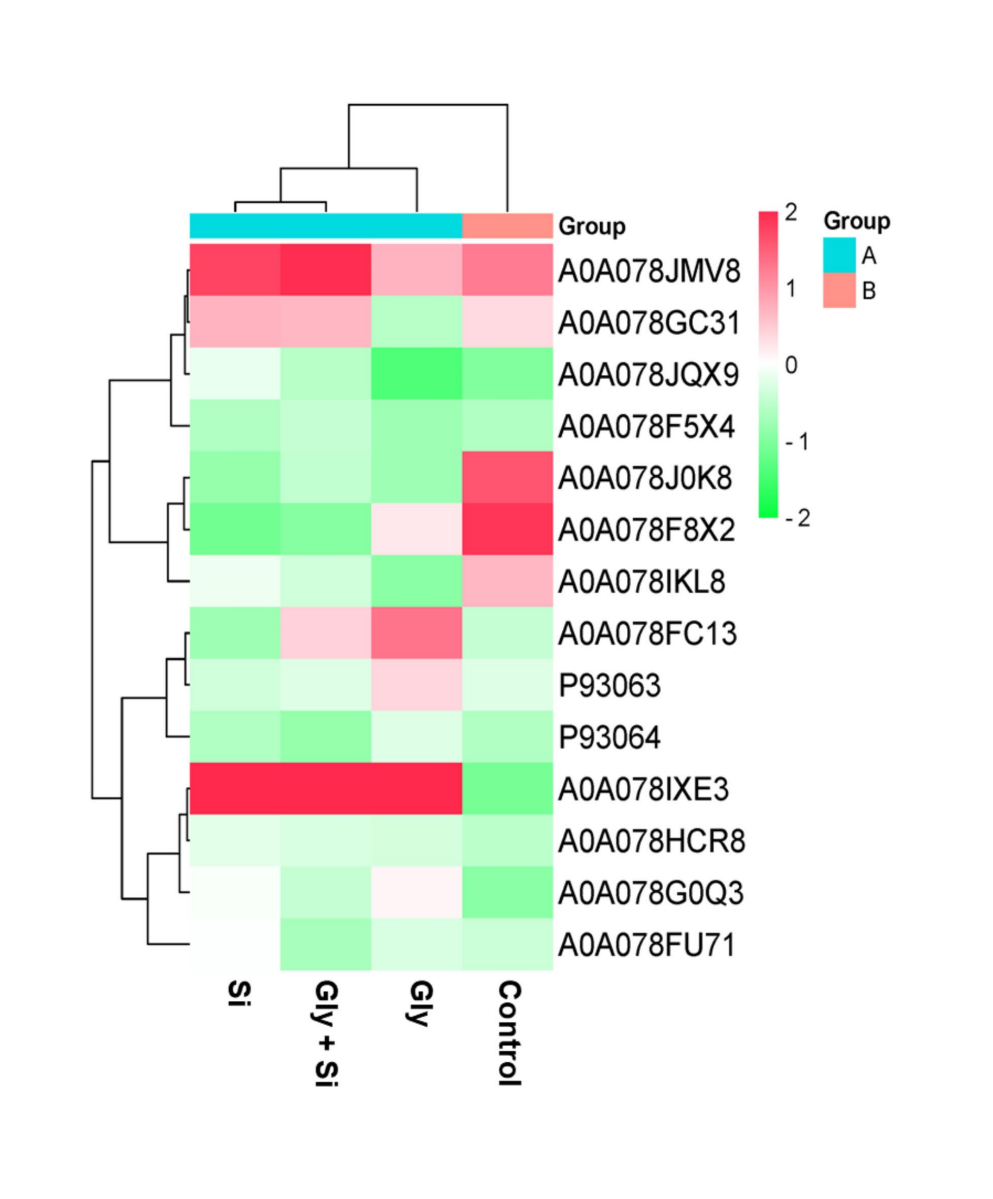
Heatmap of differentially abundant candidate proteins. The zero (0) indicates the neutral or no significant changes, while 2 value indicate the highest significant upregulation, -2 value indicates the lowest significant downregulation of candidate proteins. Abbreviation, Gly, glyphosate; Si, silicon.

We identified a total of 2004 differentially abundant common proteins across the control, Gly, Gly + Si, and Si groups (Fig. 5B). A Venn diagram was used to display the protein abundance patterns, highlighting the upregulated and downregulated DAPs for thorough understanding (Fig. 5C, Suppli. Table S3). Particularly, the commonly identified proteins were 16 DAPs among C vs. Gly, C vs. Gly + Si, and C vs. Si treatment groups. Gly stress induced a total of 75 proteins, while Gly + Si altered 106 DAPs in *B. napus*. The quantified protein profile differences were visually displayed using a heatmap. These proteins were classified according to their functions within cellular components, molecular functions, and biological processes (Fig. 6).

### GO analysis of commonly identified proteins (CIPs)

**Fig. 7:**
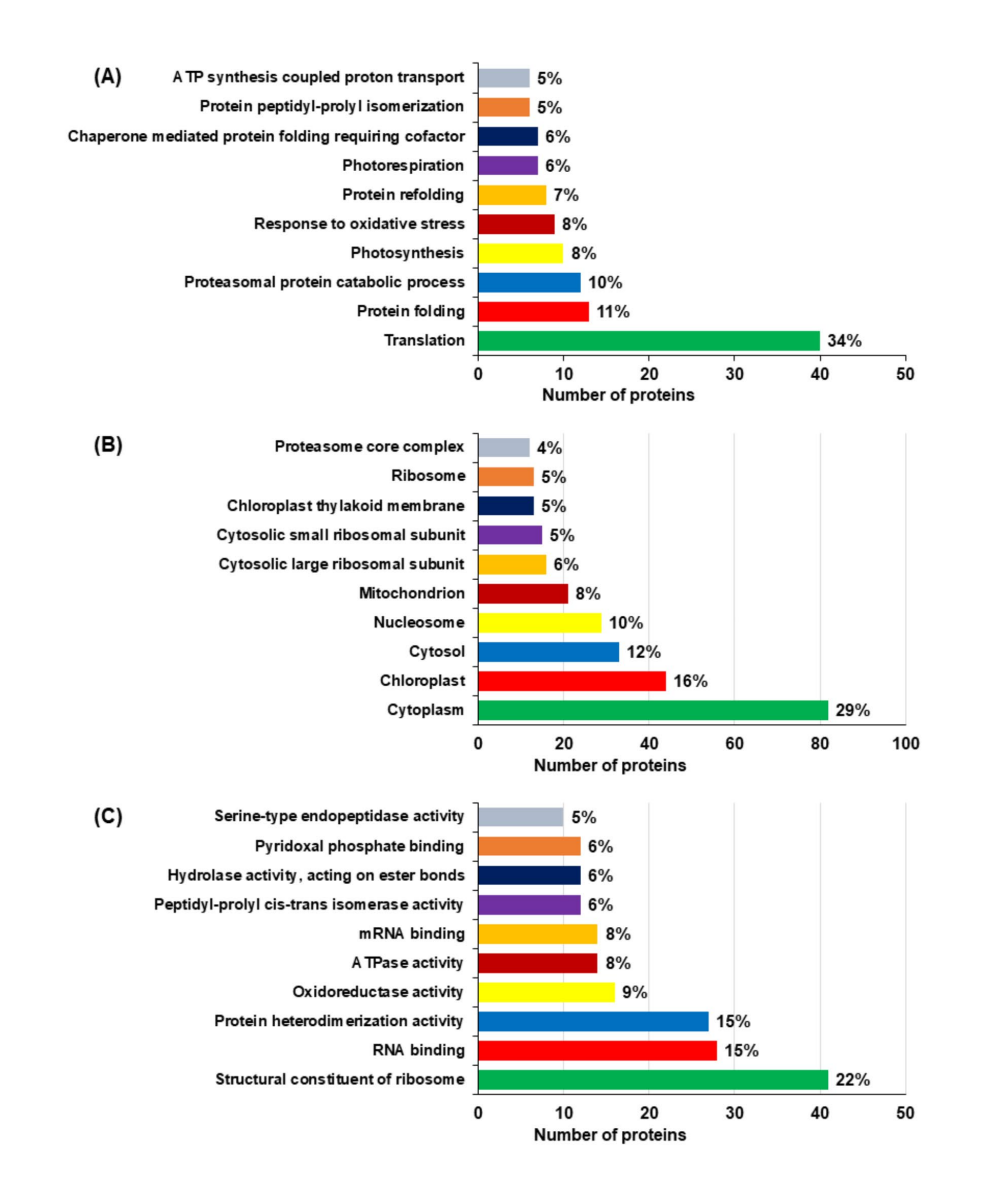
Gene ontology (GO) function analyses in *Brassica napus* leaf samples. (**A**) biological process, (**B**) cellular component, and (**C**) molecular function of the common identified proteins (CIPs) in *Brassica napus* seedlings in response to Si-mediated Gly stress using DAVID bioinformatics platform.

Biological processes, molecular functions and cellular components of frequently discovered proteins were displayed by Gene Ontology (GO) findings (Fig. 7A-C). In the category of biological process, proteins were associated with some functions, including translation (34%), protein folding (11%), proteasomal protein catabolic process (10%), Photosynthesis (8%), response to oxidative stress (8%), protein refolding (7%), photorespiration (6%), chaperone mediated protein folding requiring cofactor (6%), protein peptidyl-prolyl isomerization 5%) and ATP synthesis coupled proton transport (5%) (Fig. 7A, Suppli. Table S4). Likewise, in cellular component analyses category, the proteins showed the significant association, including cytoplasm (29%), chloroplast (16%), cytosol (12%), nucleosome (10%), mitochondrion (8%), cytosolic large ribosomal subunit (6%), cytosolic small ribosomal subunit (5%), chloroplast thylakoid membrane (5%), ribosome (5%) and proteasome core complex (4%) (Fig. 7B, Suppli. Table S4). In molecular function category, the identified proteins were associated with some functions, including structural constituent of ribosome (22%), RNA binding (15%), protein heterodimerization activity (15%), oxidoreductase activity (9%), ATPase activity (8%), mRNA binding (8%), peptidyl-prolyl cis-trans isomerase activity (6%), hydrolase activity, acting on ester bonds (6%), pyridoxal phosphate binding (6%), and serine-type endopeptidase activity (5%) (Fig. 7C, Suppli. Table S4).

The result of GO analysis showed the commonly identified differentially abundant proteins were significantly affected by Gly stress. According to the DAVID Bioinformatics analysis, a total of 2004 proteins exhibited alterations with 43 KEGG pathways correspondence (Suppli. Table S2). These proteins were associated with various metabolic pathways, including general metabolic pathways (bna01100) involving 817 proteins, carbon metabolism (bna01200) involving 214 proteins, amino acid biosynthesis (bna01230) involving 142 proteins, ribosome function (bna03010) involving 279 proteins, and secondary metabolite biosynthesis (bna01110) involving 419 proteins (Suppli. Table S2).

### GO analysis of differentially abundant proteins (DAPs)

**Fig. 8:**
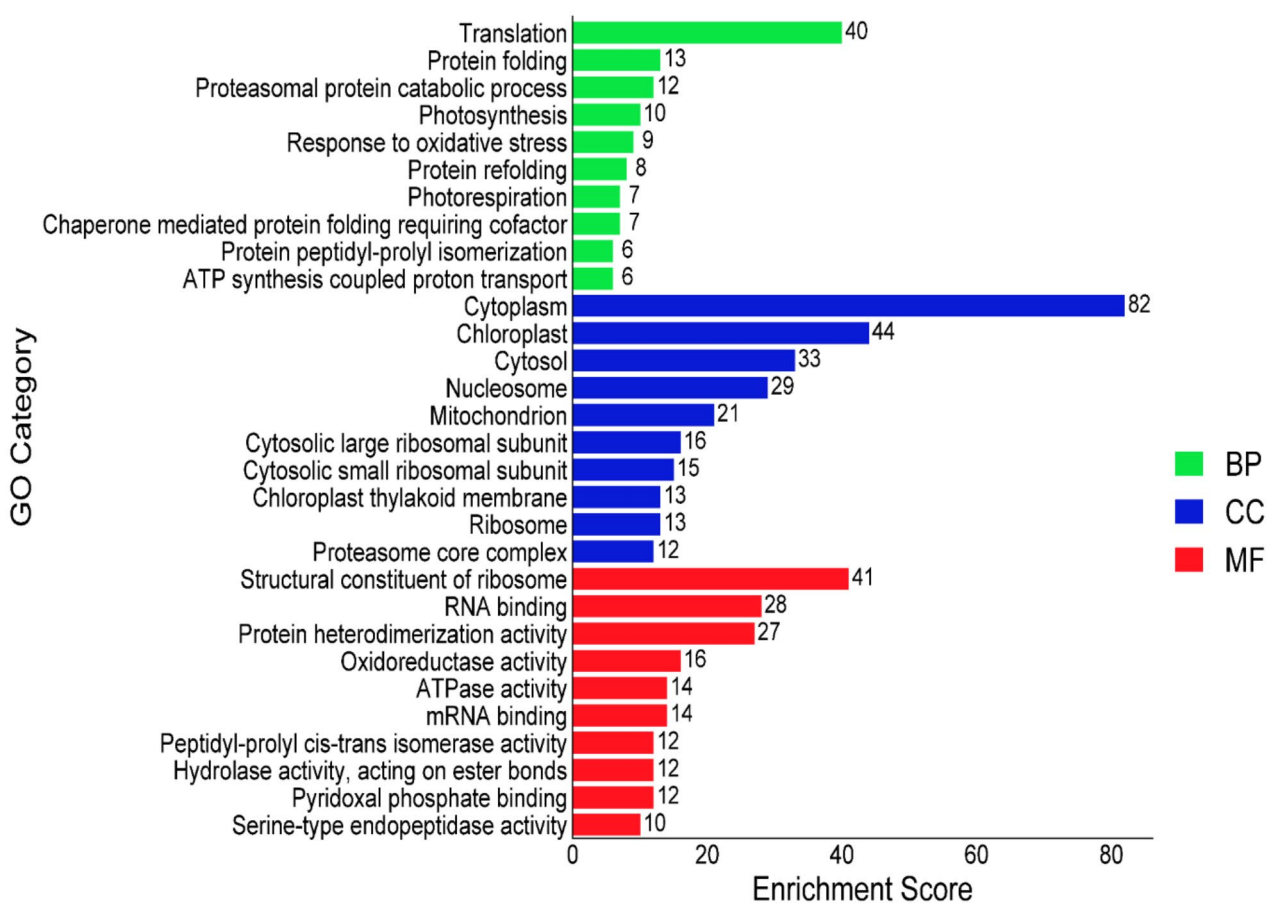
The GO functions of differentially abundant proteins (DAPs) involved in different physiological process in *Brassica napus* leaves in response to Gly and Si. Abbreviation, BP, biological process; CC, cellular component; and MF, molecular function. The bar chart was constructed through the DAVID bioinformatics platform.

The relation of DAPs to various biological process were revealed through GO analysis (Suppli. Table S1). In the category of biological process, the DAPs were associated with some processes, including translation (40 DAPs), protein folding (13 DAPs), proteasomal protein catabolic process (12 DAPs), photosynthesis (10 DAPs), response to oxidative stress (9 DAPs), protein refolding (8 DAPs), and photorespiration (7 DAPs). (Fig. 8, Suppli. Table S5). In cellular component category, cytoplasm (82 DAPs), chloroplast (44 DAPs), cytosol (33 DAPs), nucleosome (29 DAPs), mitochondrion (21 DAPs), cytosolic large ribosomal subunit (16 DAPs), cytosolic small ribosomal subunit (15 DAPs), chloroplast thylakoid membrane (13 DAPs) and ribosome (13 DAPs) displayed the leading categories (Fig. 8, Suppli. Table S5). In the category of molecular functions, GO terms of DAPs displayed important enrichment, including structural constituent of ribosome (41 DAPs), RNA binding (28 DAPs), protein heterodimerization activity (27 DAPs), oxidoreductase activity (16 DAPs), ATPase activity (14 DAPs), mRNA binding (14 DAPs), peptidyl-prolyl cis-trans isomerase activity (12 DAPs), hydrolase activity, acting on aster bonds (12 DAPs), and pyridoxal phosphate binding (12 DAPs) (Fig. 8, Suppli. Table S5).

### KEGG pathways of CIPs and DAPs

**Fig. 9:**
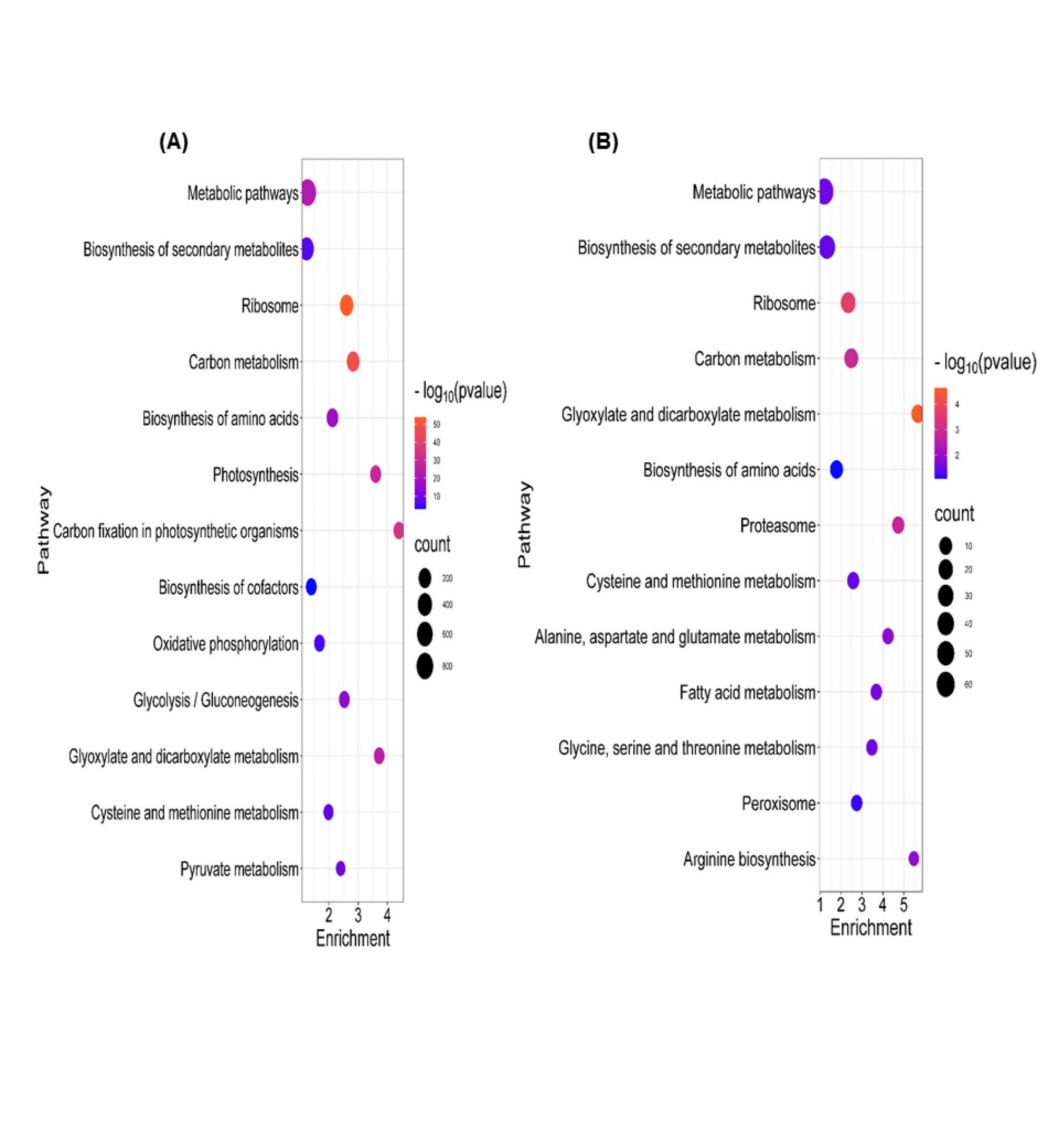
Dot plot of the KEGG pathway enrichment analysis for (**A**) common identified proteins (CIPs) and (**B**) differentially abundant proteins (DAPs) in *Brassica napus* seedlings in response to Si-mediated Gly stress. The horizontal axis represents the enrichment rate of the input proteins in the pathway, while the vertical axis represents the name of pathways. The color scale indicates different thresholds of the p-value, and the size of the dot indicates the number of proteins corresponding to each term. The bubble map was constructed through the Science and Research Plot platform (https://tinyurl.com/pve3dtnw).

The impact of Gly stress on leaf metabolism in *B. napus* seedlings was examined by constructing a potential metabolic pathway using the KEGG database (http://www.kegg.jp/; accessed on January 24, 2024). DAPs and CIPs were the key components for developing this pathway. The dot plot visualized the KEGG pathways with the highest enrichment (Fig. 9A, B).

In all treatment groups of CIPs, the KEGG pathways that showed the highest enrichment were identified as follows: metabolic pathways (bna01100) with 817 proteins, biosynthesis of secondary metabolites (bna01110) with 419 proteins, ribosome (bna03010) with 279 proteins, carbon metabolism (bna01200) with 214 proteins, biosynthesis of amino acids (bna01230) with 142 proteins, photosynthesis (bna00195) with 96 proteins, carbon fixation in photosynthetic organisms (bna00710) with 90 proteins, biosynthesis of cofactors (bna01240) with 87 proteins, oxidative phosphorylation (bna00190) with 85 proteins, glycolysis/gluconeogenesis (bna00010) with 84 proteins, glyoxylate and dicarboxylate metabolism (bna00630) with 80 proteins, cysteine and methionine metabolism (bna00270) with 67 proteins, and pyruvate metabolism (bna00620) with 62 proteins. (Suppli. Table S2).

In all DAPs treatment groups, the pathways that showed the highest enrichment were identified as follows: metabolic pathways (bna01100) with 69 proteins, biosynthesis of secondary metabolites (bna01110) with 41 proteins, ribosome (bna03010) with 23 proteins, carbon metabolism (bna01200) with 17 proteins, glyoxylate and dicarboxylate metabolism (bna00630) with 11 proteins, biosynthesis of amino acids (bna01230) with 11 proteins, proteasome (bna03050) with 8 proteins, cysteine and methionine metabolism (bna00270) with 8 proteins, alanine, aspartate and glutamate metabolism (bna00250) with 6 proteins, fatty acid metabolism (bna01212) with 6 proteins, glycine, serine and threonine metabolism (bna00260) with 6 proteins, peroxisome (bna04146) with 6 proteins, and arginine biosynthesis (bna00220) with 5 proteins (Suppli. Table S6).

### Protein network

**Fig. 10:**
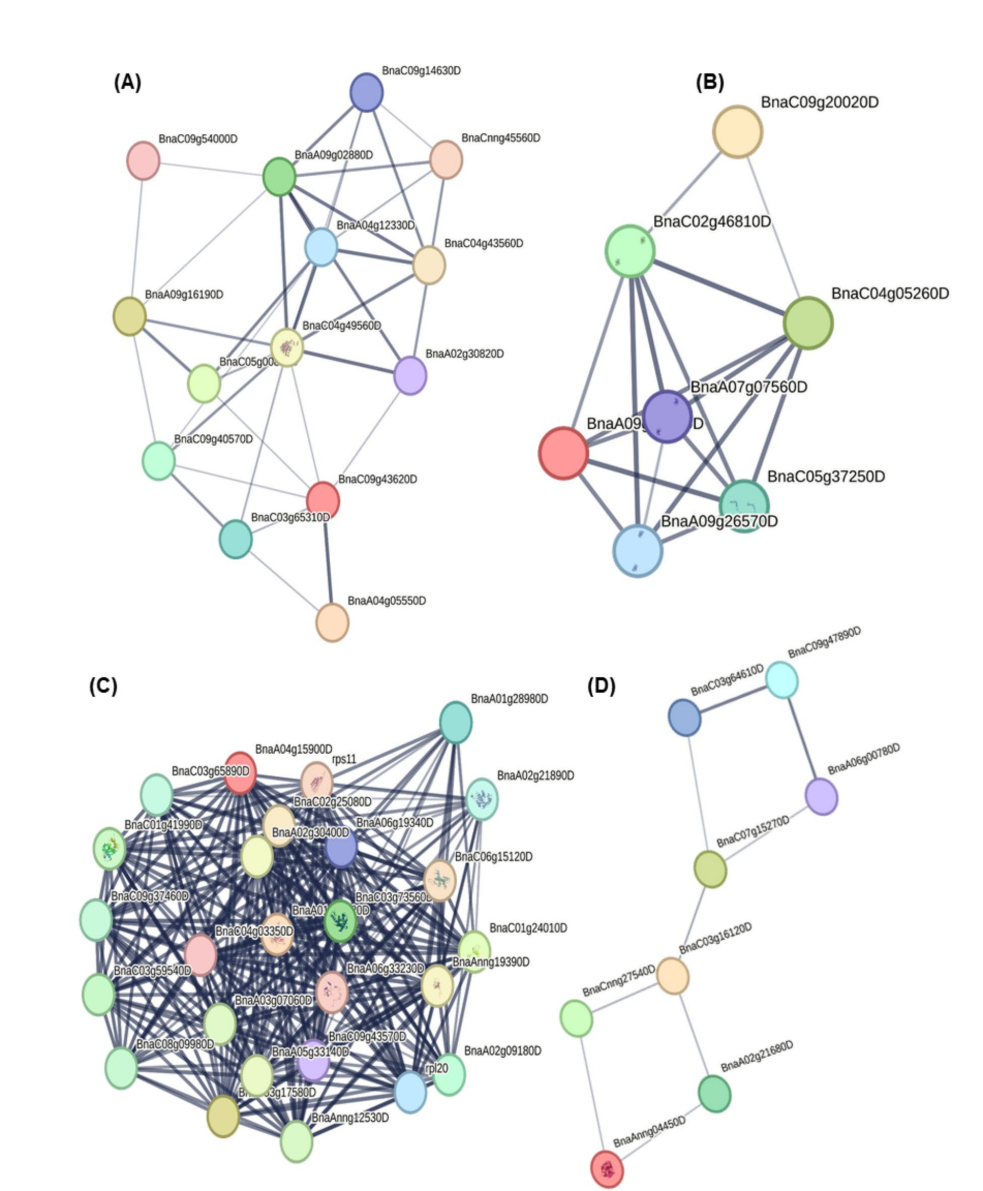
Protein interactome analysis of differentially abundant proteins (**A**) energy and metabolism, (**B**) photosynthesis, (**C**) signal transduction and (**D**) antioxidant, sulfur assimilation and herbicide tolerance related proteins identified in leaves of *Brassica napus* seedlings exposed to Si-mediated Gly stress.

**Fig. 11:**
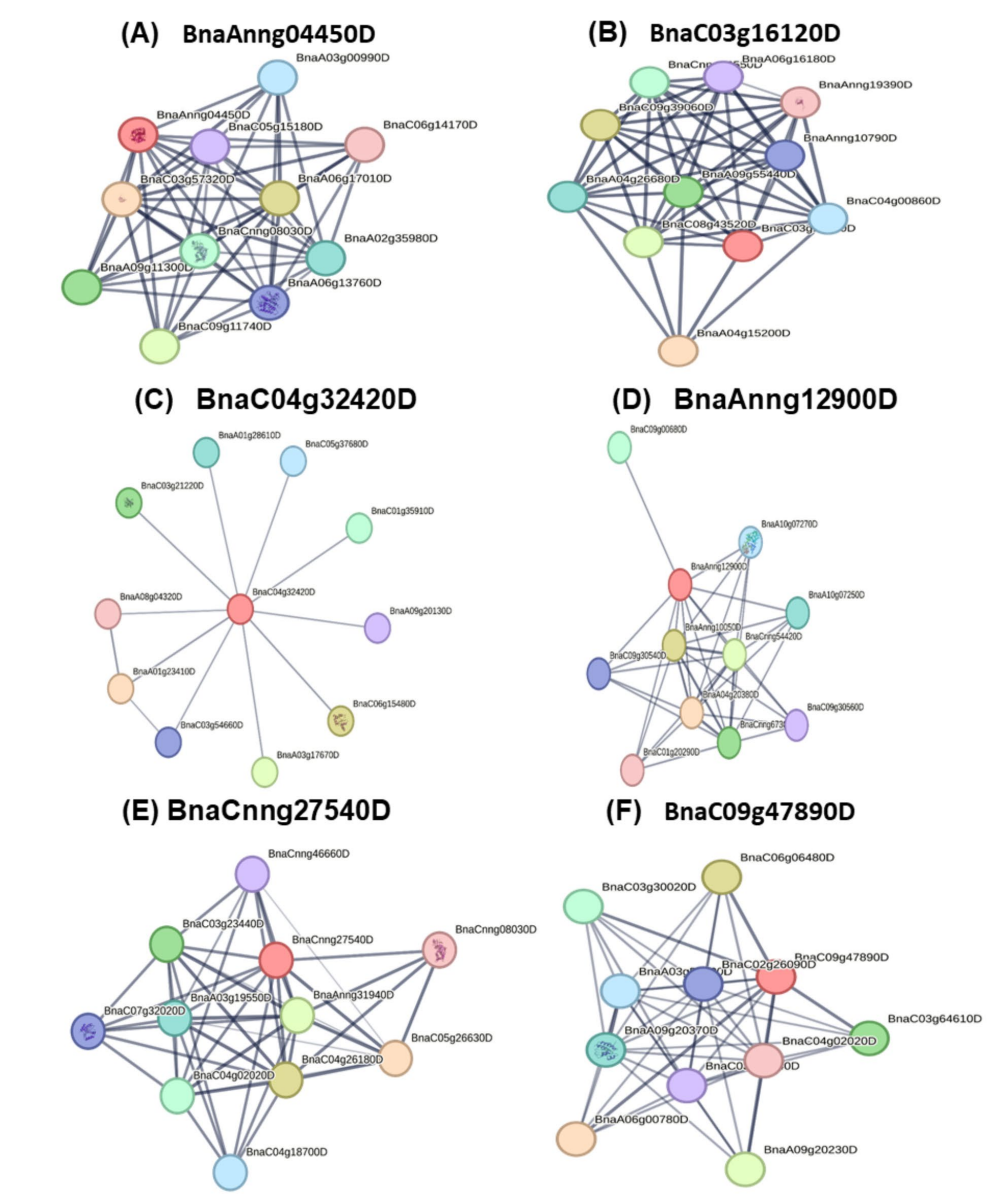
Protein-protein interaction of candidate proteins involving antioxidant, sulfur assimilation and herbicide tolerance processes in *Brassica napus*. (**A**) L-ascorbate peroxidase (A0A078HFK7, BnaAnng04450D), (**B**) Superoxide dismutase (A0A078H5C3, BnaC03g16120D), (**C**) glutaredoxin-C2 (A0A078HHH4, BnaC04g32420D), (**D**) peroxidase (A0A078IWR4, BnaAnng12900D), (**E**) glutathione peroxidase 2 (A0A078IXA9, BnaCnng27540D) and (**F**) peptide methionine sulfoxide reductase A3 (A0A078JHR9, BnaC09g47890D).

Interactions between proteins offer crucial biological insights into the processes occurring within *B. napus*. The analysis of these interactions was facilitated by the tool STRING, which categorized the proteins based on their functions. Within the category of energy-metabolism-related proteins (Table 1), the protein ATP synthase subunit O (BnaC09g43620D, A0A078F9W7) was found to interact within a network that A0A078DNQ5, A0A078FXT7, A0A078FR19, A0A078GFM5, A0A078H5Q9 and A0A078FQR6 (Fig. 10A). The photosynthesis category (Table 1) exhibited that photosystem I reaction center subunit II (BnaA09g01080D, A0A078G9Q8) sharing the protein network with A0A078J8I6, A0A078G7F6, A0A078I263, A0A078GY42 and A0A078GLT1 (Fig. 10B). The category of signal transduction (Table 1) displayed a significant interactions, where the protein (BnaA04g15900D, A0A078H195) exhibited a protein network sharing with A0A078FWF3, A0A078IT30, A0A078HEE7, A0A078HCR8, A0A078F7P2, A0A078FXI3, A0A078IMT1, A0A078F1F5, A0A078FJL5, A0A078GTV5, A0A078F3D0 and D1L8S1 (Fig. 10C). In protein categories (Table 1) antioxidant, sulfur assimilation and herbicide tolerance, the *L-ascorbate peroxidase* (BnaAnng04450D, A0A078HFK7) protein showed a share network with *GPX 2* (BnaCnng27540D, A0A078IXA9), *superoxide dismutase* (BnaC03g16120D, A0A078H5C3*)*, and probable *phospholipid hydroperoxide GPX 6* (BnaA02g21680D, A0A078GL08) (Fig. 10D). Interactome of six candidate proteins related with sulfur assimilation and herbicide tolerance, and antioxidant, including *L-ascorbate peroxidase* (A0A078HFK7, BnaAnng04450D), *superoxide dismutase* (A0A078H5C3, BnaC03g16120D), *glutaredoxin-C2* (A0A078HHH4, BnaC04g32420D), *peroxidase* (A0A078IWR4, BnaAnng12900D), *GPX 2* (A0A078IXA9, BnaCnng27540D) and *peptide methionine sulfoxide reductase A3* (A0A078JHR9, BnaC09g47890D) were shared protein networks by being mapped and analyzed within different protein interaction networks. This approach facilitated the identification of key interactions and functional roles across multiple biological contexts (Fig. 11A-F).

**Table 1:** List of differentially abundant proteins (DAPs) identified in *Brassica napus* leaves in response to gly and Si using LC MS/MS mass spectrometry.

Uniprot accession	Protein name	Gene accession	Coverage (%)	Protein score	Fold change		
					T1/CK	T2/CK	T3/CK
**Energy and metabolism-related proteins**							
A0A078F9W7	ATP synthase subunit O	BnaC09g43620D	27.9	112	0.641914	0.308654	0.515482
A0A078DNQ5	ATP synthase subunit d	BnaA04g05550D	39.9	221	0.610627	0.07293	0.532053
A0A078HBM9	Sucrose-phosphate synthase	BnaC09g37470D	5.2	51	1.160603	1.054256	0.368837
A0A078JFK9	Glucose-6-phosphate isomerase	BnaA09g16190D	21.1	137	1.110806	0.859891	0.398182
A0A078FQR6	Pyruvate dehydrogenase E1 component subunit alpha	BnaC05g00810D	30.1	389	0.278459	0.369308	0.501324
A0A078FHB3	NADP-dependent D-sorbitol-6-phosphate dehydrogenase	BnaA09g43270D	10.7	38	0.560104	0.477702	0.576195
A0A078G5J5	Acetyl-CoA acetyltransferase, cytosolic 1	BnaA09g02880D	8.7	88	1.560391	1.027869	1.465700
A0A078FR19	2-hydroxyacyl-CoA lyase	BnaC09g40570D	12.3	115	1.543537	1.151831	0.941912
A0A078I1T6	3‘(2’),5’-bisphosphate nucleotidase	BnaA06g23120D	16.6	195	1.551473	1.401666	1.308774
A0A078FXT7	Isocitrate dehydrogenase [NAD] regulatory subunit 1, mitochondrial-like	BnaC03g65310D	11	113	1.644654	1.450731	1.134525
A0A078J7I1	Probable NAD(P)H dehydrogenase (quinone) FQR1-like 2	BnaAnng18230D	4.2	51	2.130197	1.023216	1.279924
A0A078HTX7	Ferredoxin-thioredoxin reductase, variable chain-like	BnaC02g41630D	20.3	207	1.147394	0.556879	1.243849
A0A078FU33	Acetyl-coenzyme A synthetase, chloroplastic/glyoxysomal	BnaA04g12330D	4.7	66	0.940429	0.552378	0.889766
A0A078JN33	Glucose-1-phosphate adenylyltransferase	BnaC09g54280D	20	161	0.677495	0.652565	0.898576
A0A078GX98	Ferredoxin	BnaA01g18870D	36.1	346	0.675095	0.260915	0.741719
A0A078H0E1	ATP-dependent Clp protease proteolytic subunit-related protein 3	BnaCnng06590D	8.6	142	0.417604	0.808942	0.739474
A0A078I6S3	Ferredoxin, leaf L-A-like	BnaCnng11890D	60.8	493	0.576922	1.184970	1.457765
A0A078FLD4	ATP-dependent Clp protease proteolytic subunit	BnaC05g01290D	23.9	243	0.627616	0.844326	0.937172
A0A078GA43	Alternative NAD(P)H-ubiquinone oxidoreductase C1	BnaC09g47290D	11	81	0.535182	0.587395	1.150194
A0A078FUD9	NAD(P)H-quinone oxidoreductase subunit U	BnaC09g36720D	6.5	54	0.417060	0.634000	1.045862
A0A078ICZ4	1,4-dihydroxy-2-naphthoyl-CoA synthase	BnaC09g14630D	14.5	122	0.650433	0.614697	0.762897
A0A078JJZ9	Glucose-6-phosphate 1-dehydrogenase	BnaCnng48180D	12	175	0.532154	0.641487	0.965326
A0A078F3 × 9	Acetyl-CoA carboxytransferase	BnaA03g17570D	23.9	595	0.508116	0.555084	0.801322
A0A078IZH1	Phosphoglucomutase (alpha-D-glucose-1,6-bisphosphate-dependent)	BnaC02g44120D	16.3	141	0.648889	0.656212	0.801299
A0A078GTL5	NADPH-protochlorophyllide oxidoreductase	BnaA03g48610D	28.3	1331	0.510446	0.606909	1.297102
A0A078J698	Ribulose bisphosphate carboxylase/oxygenase activase	BnaC06g43890D	18.8	148	0.598431	0.549122	0.814076
A0A078H5Q9	ATP-citrate synthase beta chain protein 2	BnaA02g30820D	27.1	420	0.563960	0.590482	0.666005
A0A078FEZ7	Phosphoglucomutase (alpha-D-glucose-1,6-bisphosphate-dependent)	BnaC06g31970D	11.5	128	0.336701	0.301114	0.707119
A0A078IUF6	4-hydroxy-3-methylbut-2-en-1-yl diphosphate synthase (ferredoxin)	BnaC09g54000D	40.1	757	0.646842	0.677358	0.745123
A0A078IKT4	NAD(P)H-quinone oxidoreductase subunit M	BnaC03g61160D	20.9	74	0.572738	1.050565	0.787579
A0A078GAK7	Probable NADH dehydrogenase [ubiquinone] 1 alpha subcomplex subunit 12	BnaC04g49930D	12.6	56	1.516589	1.662329	1.532316
A0A078JE46	3-hydroxyisobutyryl-CoA hydrolase	BnaCnng45560D	19.2	228	1.409853	1.666521	1.150921
A0A078GEI0	3-ketoacyl-CoA thiolase 2, peroxisomal-like	BnaC04g43560D	18.4	251	1.576170	1.540472	1.301873
A0A078GFM5	Citrate synthase	BnaC04g49560D	29.3	431	1.718077	2.309301	0.757696
A0A078HMR7	Ferredoxin–NADP reductase, chloroplastic	BnaC09g21770D	6.6	76	2.784496	3.937910	2.047532
**Photosynthesis-related proteins**							
A0A078GDB8	Pyruvate dehydrogenase E1 component subunit alpha-1	BnaA01g22040D	21.9	279	1.503392413	1.095588	0.979475
A0A078H395	Acetyltransferase component of pyruvate dehydrogenase complex	BnaC06g07040D	4.9	163	1.529129	1.144697	0.987798
A0A078G9Q8	Photosystem I reaction center subunit II	BnaA09g01080D	60	731	0.779631	0.524350	1.207470
A0A078JQI2	Ribulose bisphosphate carboxylase small subunit	BnaCnng55860D	60.8	2977	0.687302	0.59090	0.935430
A0A078FCT5	Cytochrome b5	BnaC04g12100D	21.8	32	0.582401	1.026269	1.014617
A0A078GHQ4	Light-harvesting complex-like protein 3 isotype 2	BnaC09g20020D	12.9	65	0.630690	0.849381	1.319439
A0A078G7F6	Chlorophyll a-b binding protein	BnaC04g05260D	44.6	643	0.612507	0.803099	1.146516
A0A078F191	RuBisCO large subunit-binding protein subunit beta	BnaA05g13740D	56.8	1586	0.654017	0.754457	0.875406
A0A078JWT1	Carboxypeptidase	BnaCnng66490D	4.5	91	0.560883	0.615016	1.027021
A0A078IQK6	psbP domain-containing protein 2	BnaA04g16500D	9.3	56	0.413269	0.453155	0.789982
A0A078GUT8	Protochlorophyllide reductase B	BnaC01g19630D	58.7	2075	0.466504	0.471211	1.074177
A0A078J638	Photosystem II stability/assembly factor HCF136	BnaC07g49760D	38.7	609	0.651477	0.648676	0.762717
A0A078IBE5	Cytochrome c oxidase subunit 6b-1	BnaA07g10470D	28.1	197	1.141994	1.215388	1.806969
A0A078J8I6	Chlorophyll a-b binding protein CP26	BnaC02g46810D	52	1277	0.618674	1.027023	1.591433
A0A078GY42	Photosystem I reaction center subunit VI-1	BnaC05g37250D	47.6	356	0.935938	1.026269	1.997100
A0A078GLT1	Chlorophyll a-b binding protein 1	BnaA09g26570D	67.4	1663	0.845850	0.967379	1.529286
A0A078I263	Chlorophyll a-b binding protein 1	BnaA07g07560D	67.4	1535	0.845850	0.967379	1.529286
A0A078F5S2	Cytochrome b-c1 complex subunit Rieske	BnaC09g43660D	14.8	78	2.858618	3.551948	5.188301
**Signal transduction**							
A0A078H195	Ribosomal protein	BnaA04g15900D	17.9	357	0.936630	0.301479	0.542141
A0A078G6 × 0	30 S ribosomal protein S3	BnaA01g34120D	58.6	272	0.413802	0.300090	0.465979
A0A078GH13	40 S ribosomal protein S3-2-like	BnaC03g17580D	45.4	541	0.420976	0.633565	0.500272
A0A078G4M4	50 S ribosomal protein L9	BnaC01g24010D	42.3	345	0.363187	0.106646	0.505546
A0A078IQV6	Peptidylprolyl isomerase	BnaA03g57740D	19	160	0.754682	0.878080	0.593926
A0A078CAM5	40 S ribosomal protein S5-1	BnaC03g73560D	29	259	0.811626	0.423112	0.523401
A0A078G7Z8	Peptidylprolyl isomerase	BnaA02g09180D	38.5	583	0.428816	0.415404	0.613298
A0A078FTA7	Peptidyl-prolyl cis-trans isomerase NIMA-interacting 4	BnaA09g28980D	25.9	118	0.611649	0.672394	0.451239
A0A078G3Y5	30 S ribosomal protein S20	BnaA01g28980D	17.1	154	0.727015	0.289791	0.966337
D1L8R1	Ribosomal protein L20	rpl20	29.1	155	0.878813	0.385762	0.793634
A0A078GTV5	50 S ribosomal protein L22	BnaA06g19340D	28.8	343	0.958983	0.624092	0.973985
A0A078F9 × 4	50 S ribosomal protein L10	BnaC09g43570D	21.6	307	0.463351	0.612582	0.865896
A0A078FXI3	60 S ribosomal protein L7-3	BnaC04g03350D	31.8	250	0.651839	0.287923	0.693873
A0A078J2Z4	Aquaporin PIP2-1	BnaCnng31040D	11.5	143	0.705092	0.641418	0.804387
A0A078FGQ0	50 S ribosomal protein L15	BnaA06g33230D	39.5	679	0.591781	0.649133	0.957670
D1L8S1	Ribosomal protein S11	rps11	34.1	240	0.409063	0.615914	1.131517
A0A078F3D0	50 S ribosomal protein L17	BnaC06g15120D	20.6	92	0.276407	0.408465	1.137502
A0A078FJL5	50 S ribosomal protein L13	BnaC02g25080D	38.3	685	0.771884	0.539970	0.945665
A0A078JCZ3	30 S ribosomal protein S9	BnaAnng19390D	32.1	287	0.674137	0.438083	1.063720
A0A078F1F5	60 S ribosomal protein L13a-4	BnaA02g30400D	26.7	488	0.940910	0.542686	0.947710
A0A078GAJ5	GTP-binding protein SAR1A	BnaA09g00560D	22.3	114	0.653454	0.716521	1.066103
A0A078GNF2	Peptidyl-prolyl cis-trans isomerase-like	BnaC09g09060D	69	1005	0.630977	0.771292	0.958601
A0A078IHA7	40 S ribosomal protein S16-3-like	BnaA05g33140D	52.1	138	0.751221	0.582845	0.814145
A0A078IMT1	40 S ribosomal protein S16-3	BnaA03g07060D	52.1	179	0.751221	0.582845	0.814145
A0A078IUM5	50 S ribosomal protein L1	BnaAnng12530D	40.4	740	0.856801	0.648034	1.004073
A0A078IT30	40 S ribosomal protein S7	BnaC01g41990D	41.9	638	0.853907	0.650349	1.402254
A0A078HCR8	60 S ribosomal protein L18a-2-like	BnaC03g59540D	26.4	82	1.609555	0.843797	1.047634
A0A078GP33	Chaperone protein ClpB3	BnaC09g42450D	4.8	85	1.7074686	1.046612	0.655224
A0A078FY34	Co-chaperone protein p23	BnaC03g30910D	4.6	37	1.374372	1.500503	1.286792
A0A078FDD6	Peptidyl-prolyl cis-trans isomerase CYP28	BnaC08g08050D	18.6	115	1.183340	2.637557	1.474462
A0A078JIQ0	Peptidyl-prolyl cis-trans isomerase FKBP18	BnaCnng50370D	5.7	41	1.742753	1.594670	2.467164
A0A078HTZ3	Peptidylprolyl isomerase	BnaA01g05720D	13.5	92	1.530424	1.612151	1.240177
A0A078DMP0	Peptidyl-prolyl cis-trans isomerase CYP18-3	BnaC03g60160D	54.7	545	1.412029	1.775994	0.604640
A0A078F7P2	60 S ribosomal protein L27	BnaC08g09980D	26.6	99	1.329520	0.871083	1.792157
A0A078J6H5	Peptidyl-prolyl cis-trans isomerase	BnaA04g27460D	26	429	1.272562	1.207818	1.607132
A0A078HEE7	40 S ribosomal protein S8	BnaC09g37460D	38.4	882	1.158817	1.364160	1.613969
A0A078FWF3	40 S ribosomal protein S3a	BnaC03g65890D	32.7	210	0.935013	1.209417	1.601761
A0A078GVL1	14-3-3-like protein GF14 nu	BnaCnng05840D	23.4	103	1.457646	1.713257	2.099661
A0A078GJ98	Nucleoside diphosphate kinase III	BnaA02g21890D	23.8	194	1.475067	1.916405	1.866466
A0A078GVF8	Peptidylprolyl isomerase	BnaA06g14640D	6.5	83	2.027866	1.425783	1.857198
**Antioxidant-related proteins**							
A0A078HFK7	L-ascorbate peroxidase	BnaAnng04450D	21.4	221	0.811401	0.240271	0.715595
A0A078H5C3	Superoxide dismutase	BnaC03g16120D	7.7	79	0.575918	0.283087	0.481150
A0A078F5 × 4	Formate dehydrogenase	BnaC09g42760D	30.5	212	2.212749	2.407629	1.158634
A0A078IIB3	Heat shock 70 kDa protein 9	BnaC03g61170D	19.4	519	1.546281	1.366098	0.797890
A0A078GSE1	2Fe-2 S ferredoxin-like	BnaC05g44490D	7.6	43	0.935938	2.318608	1.754885
A0A078FU93	Glutaredoxin-C4	BnaC09g37340D	14.4	106	0.933258	1.579110	1.629717
A0A078HHH4	Glutaredoxin-C2	BnaC04g32420D	44.1	261	1.483304	1.613732	1.002576
A0A078IWR4	Peroxidase	BnaAnng12900D	17.1	233	1.006332	0.651766	0.771208
A0A078H2Z1	Catalase-1	BnaC07g15270D	19.1	239	1.290740	1.639032	1.052930
**Cell wall-related proteins**							
A0A078GI07	Eukaryotic translation initiation factor 3 subunit F	BnaC03g22380D	14.3	127	1.234423	1.811705	1.281683
A0A078F7G0	Endo-1,3; 1,4-beta-D-glucanase-like	BnaC01g30540D	27.6	70	1.256936	1.513637	0.818176
A0A078GZR9	Glucan endo-1,3-beta-glucosidase	BnaC04g24330D	28.8	266	0.998580	1.264229	1.730090
A0A078FEH1	Germin-like protein	BnaA09g39580D	13.2	88	0.933341	1.519650	1.572200
A0A078I1B1	Glucan endo-1,3-beta-glucosidase 6	BnaC09g33610D	4.6	34	1.312819	1.629957	2.011610
A0A078BXJ1	Probable glucan endo-1,3-beta-glucosidase BG3	BnaCnng07500D	8.5	113	2.334582	2.821929	2.367895
A0A078JUQ1	Xyloglucan endotransglucosylase/hydrolase	BnaCnng62600D	21	144	0.661825	0.496963	1.248429
A0A078GSK1	Xyloglucan endotransglucosylase/hydrolase protein 24-like	BnaA01g06750D	9.6	54	1.121947	0.650191	1.083272
**Herbicide tolerance and sulfur assimilation**							
A0A078IXA9	Glutathione peroxidase 2	BnaCnng27540D	12.4	57	0.399320	0.980806	0.745672
A0A078IY55	Protein disulfide isomerase-like 1–3	BnaA09g55010D	2.1	53	0.645412	0.562957	1.105768
A0A078FY06	Thiocyanate methyltransferase 1	BnaC04g03180D	40.3	253	0.769093	0.843758	0.584605
A0A078H0T9	Cysteine synthase, chloroplastic/chromoplastic-like	BnaA04g25390D	50.9	1629	0.936927	0.750959	0.626689
A0A078FXM1	Cysteine synthase, chloroplastic/chromoplastic	BnaC04g03050D	51.3	1127	1.034496	0.616173	0.644198
A0A078GL08	Probable phospholipid hydroperoxide glutathione peroxidase 6	BnaA02g21680D	14.4	87	0.791784	1.669100	1.070403
A0A078JMZ9	Glutamine synthetase cytosolic isozyme 1–3	BnaCnng57900D	19.2	311	0.927470	1.618427	0.919078
A0A078FXL6	Cysteine proteinase RD21A	BnaC06g00260D	12.7	107	1.347847	2.516433	1.463737
A0A078IFI4	Bifunctional D-cysteine desulfhydrase/1-aminocyclopropane-1-carboxylate deaminase	BnaC06g01990D	11.4	142	1.267775	1.516280	0.571718
A0A078JHR9	Peptide methionine sulfoxide reductase A3	BnaC09g47890D	5.4	58	0.935938	2.309105	1.287020
A0A078GQ38	Peptide methionine sulfoxide reductase B9	BnaC03g64610D	12.5	53	0.659267	1.332218	1.342620
A0A078GC86	Peptide methionine sulfoxide reductase B1	BnaA06g00780D	4.9	38	0.657927	0.721426	0.904724
A0A078FC13	Cystine lyase CORI3	BnaC01g15060D	29	302	2.188200	3.346658	0.936847
**Plant development-related proteins**							
A0A078FG80	Early nodulin-like protein 1	BnaA03g51730D	13.5	72	0.655443	1.029282	1.237389
A0A078H4R1	Elongation factor G	BnaC09g13110D	41.3	1648	0.625852	0.582129	0.965449
A0A078ITJ0	Eukaryotic translation initiation factor 3 subunit G	BnaCnng24840D	14.3	99	1.620657	0.959656	1.024812
A0A078HEL5	Elongation factor Ts, mitochondrial	BnaA01g07830D	44.7	1786	0.499439	0.499206	0.742859
A0A078HFL8	Nodulin-related protein 1	BnaC02g35030D	22.1	95	0.473006	1.215405	1.256836
A0A078G1A8	Early nodulin-like protein 2	BnaC01g20120D	15.1	71	2.193042	1.836736	1.521730
A0A078HAL9	Early nodulin-like protein 1	BnaA09g41080D	11.2	37	0.974623	0.643809	1.882587
**Unknown**							
A0A078GYM2	Uncharacterized protein At5g39570-like	BnaC04g31650D	18.2	275	1.364852	1.500183	1.270977
A0A078FG18	Uncharacterized BNAC06G13920D	BnaC06g13920D	29.1	91	0.681077	0.412087	0.807609
A0A078HEC4	Uncharacterized LOC106361260	BnaA08g04600D	9.6	70	0.653270	0.716320	1.279594
A0A078FEM7	Uncharacterized LOC106429057	BnaC03g70960D	11.2	74	0.858384	0.185963	0.538548
A0A078GY92	Uncharacterized BNAA03G49580D	BnaA03g49580D	9.7	115	0.596141	1.232989	0.597545
A0A078J322	Uncharacterized LOC106433592	BnaC05g49260D	13.8	369	0.221306	0.206383	0.541906
A0A078HEZ5	Uncharacterized LOC106452857	BnaA05g29710D	24.7	271	0.615216	0.525478	0.437132

## Discussion

The study offers proteome insights into the mechanisms underlying Si-mediated protection against Gly toxicity in *B. napus*. We observed Gly-induced toxicity leads to the generation of excess ROS consequently increasing the oxidative stress, and negatively affecting the morphological and physiological characteristics of the *B. napus* seedlings. However, addition of Si restored the oxidative stress. In our study, Si restored the pigments of photosynthesis as well as increased GST activity. A relationship exists between the pigments of light-harvest and efficiency of photosynthesis, which in turn enhances growth and development in *B. napus*^32^. Therefore, Si is crucial in restoring the pigments of photosynthesis in *B. napus* under Gly-toxicity. Additionally, the minimization of Gly-induced oxidative stress indicators (O_2_^•−^, H_2_O_2_) in response to Si supplementation indicates that Si actively reduces oxidative stress in plants under Gly stress. This reduction in oxidative stress is achieved through the ability of Si to enhance antioxidant activity, thereby improving plant tolerance to abiotic stress^33^.

GO and KEGG analysis are vital for proteomic studies as they provide comprehensive insights into the functional roles and pathways associated with identified proteins. The analysis of functional categories for 130 DAPs suggests their involvement in diverse biological processes, including energy and metabolism, photosynthesis, signal transduction, antioxidant, cell wall functions, herbicide tolerance and sulfur assimilation, and plant development. In the subsequent categories, we explored all mechanisms and interpretations, highlighting results that either align or differ from those observed in other plant species when compared to *B. napus*.

### Energy and metabolism-related proteins

Plants produce carbohydrates through photosynthetic CO_2_ fixation, which serve as substrates for various functions, including energy metabolism, secondary metabolism, growth, development, and stress responses. Proper regulation of photosynthesis and carbohydrate metabolism is crucial for plant growth, development, and stress adaptation, especially in a changing environment^34^. Energy production from carbohydrates is vital for supporting metabolic processes and the growth of plants. Studies have demonstrated a connection between elevated glucose storage and improved resilience to metal stress in several plant species^35^.

In this research, we identified 35 DAPs engaged in citric acid cycle and the metabolism of carbohydrate. Plants have developed intricate regulatory mechanisms to regulate glycolysis pathways and the TCA cycle, ensuring efficient energy production and metabolic adaptation during environmental stress. These pathways are fine-tuned to optimize resource utilization and maintain cellular homeostasis under abiotic stresses^36^. The increase in proteins associated with glycolysis suggests the *B. napus* seedlings can maintain critical respiration processes and produce more ATP through enhanced glycolytic activity under Gly stress. Si application enhances the ability of plants to mitigate toxic effects on cellular components through cellular homeostasis.

Proteins such as NADH dehydrogenase [ubiquinone] 1 alpha subcomplex subunit 12, NAD(P)H-quinone oxidoreductase subunit U, and pyruvate dehydrogenase E1 component subunit, which are engaged in the citric acid cycle, help maintain cellular energy production and metabolic balance under Gly stress. These results align with findings from a proteomic study on the roots of *Oenothera glazioviana* under the stress of Cu^37^. Collectively, these proteins engaged in the citric acid cycle, along with the beneficial effects of Si supplementation on antioxidant activity, metabolic pathways, and plant physiology, contribute to Gly stress tolerance in *B. napus*.

### Photosynthesis-related proteins

Plants regulate their photosynthetic capacity in response to environmental conditions, optimizing yield and overall growth and development^34^. Gly-toxicity markedly inhibits photosynthesis and plant growth by immobilizing essential micronutrients required for chlorophyll formation and photosynthesis and disrupting physiological processes and cell metabolism^38,39^. In our study, we observed unique response patterns of differentially abundant proteins (DAPs) associated with photosynthesis, particularly those involved in the Calvin cycle and the electron transport chain, under Gly stress. The detrimental effects of Gly on photosynthesis have also been documented in willow plants^38^.

The reduced abundance of leaf proteins, such as ribulose bisphosphate, indicates that energy metabolism and carbon fixation processes are significantly impaired in *B. napus* Gly toxicity, likely due to diminished gas exchange capacity^40^. These findings indicate that Gly toxicity impairs photosynthetic machinery. Additionally, our study suggests that Gly or Gly + Si induced proteomic regulations did not show similar abundance patterns. However, physiological analysis confirmed that Si restored the content of photosynthetic pigments.

Thus, the proteomic alterations induced by Gly + Si suggest that Si actively responds even under Gly stress. The findings suggest that increased energy generation is necessary for *B. napus* under Gly stress in order to activate carbon fixation. The higher abundance of these potential proteins under Gly stress indicates that they may have a role in supporting *B. napus* plants by encouraging better growth and development.

### Signal transduction

Signaling pathway is crucial for mobilizing defense mechanisms against toxic substances in the plants^41^. This proteomic study examines Gly-associated signal alterations and the metabolism of differentially abundant proteins. We discovered that the peptidyl-prolyl cis-trans isomerase CYP28 is upregulated, while CYP18-3 is downregulated. Both of these enzymes play a crucial role in speeding up the cis-trans isomerization of proline imidic peptide bonds in oligopeptides, aiding in protein folding. Stress signals triggered by protein kinases lead to changes in gene expression, resulting in the upregulation of CYP28 and downregulation of CYP18-3, contributing to Gly stress tolerance in *B. napus*. Additionally, we identified an upregulated GTP-binding protein SAR1A. The upregulation of SAR1A by Gly treatment suggests it functions as a molecular switch in signal transduction cascades, contributing to Si-mediated Gly stress tolerance in *B. napus*. Comparable results were found in a proteomic study of glyphosate-induced oxidative stress in rice leaves^42^.

### Antioxidant-related proteins

Several stress-related and defensive proteins, along with vital antioxidant enzymes, play crucial roles in defending plant mechanisms, and enhancing stress tolerance^43^. In this current study, nine DAPs were identified under the stress of Gly. Among these, one SOD, one CAT, and one APX showed notable upregulation in response to Gly and Si exposure. SOD and APX are key players in ROS detoxification in plant cells. SOD, the first enzyme in the detoxification process, converts superoxide anion (O_2_^•−^) to H_2_O_2_, while APX reduces H_2_O_2_ to water using ascorbic acid as an electron donor^44^. Comparable results were found in a proteomic study of glyphosate-induced oxidative stress in rice leaves^42^.

Two possible ways that Si reduces abiotic stress are by controlling ROS levels and enhancing antioxidant metabolism^45^. Our results revealed that Gly induced ROS were successfully regulated by enhanced activities of DAPs and key antioxidants (SOD, APX, CAT). This indicates that the antioxidant defense system was completely operational under Gly stress, further enhancing Gly tolerance in *B. napus* through exogenous Si supplementation.

### Cell wall-related proteins

Cell wall and cytoskeleton related proteins cause rapid changes due to Gly exposure. The cell wall acts as the primary mechanism against abiotic stressors such as Gly toxicity, acting as a barrier and undergoing modifications when under stress. We identified eight differentially abundant proteins (DAPs) under Gly stress. Among these proteins, the xyloglucan endotransglucosylase/hydrolase protein family, which cleaves and reconnects xyloglucan molecules, plays a crucial role in cell wall synthesis, reconstruction, and stress resistance. This supports findings in poplar plants that show tolerance to abiotic stress, such as salt stress^46^. Our results indicate that Si-mediated tolerance to Gly stress involves the processing of proteins associated with the cell wall.

### Herbicide tolerance and sulfur assimilation

Sulfur (S) is crucial for regulating plant tolerance to glyphosate^47^. In our *B. napus* proteome research, we identified thirteen key proteins involved in herbicide tolerance and sulfur assimilation processes, which enhance development and growth of *B. napus* under Gly toxicity. We noted increased levels of proteins associated with sulfur assimilation, which aid in incorporating sulfur into compounds such as methionine and GSH, thereby enhancing Gly tolerance in *B. napus*. The role of Si in mitigating Gly toxicity has also been observed in tomato plants, highlighting its protective effects against Gly-induced stress^5^. Among these proteins, three peptide methionine sulfoxide reductase proteins, which catalyze the reduction of methionine sulfoxide to methionine, play a protective role against oxidative stress^48^. In this current study, the minimization of H_2_O_2_ and O_2_^•−^ in response to Si supplementation suggests that involvement of Si in Gly tolerance is linked to its availability in *B. napus*. Additionally, the significant upregulation of these three DAPs indicate the role of Si in facilitating sulfur-mediated Gly tolerance in *B. napus*.

### Plant development-related proteins

We identified seven DAPs associated with plant growth and development. Notably, early nodulin-like protein 1 was significantly upregulated, which plays a critical role in regulating translation processes within plants. This protein plays the crucial role under stress responses, as validated by a combined transcriptome and proteome approach in pigeon pea^49^. In our investigation, this heightened synthesis of protein, activated by silicon, enhances the cellular defensing ability against Gly toxicity and promotes overall growth of the plants.

### Unknown proteins

Several unknown proteins, such as uncharacterized protein At5g39570-like, uncharacterized BNAA03G49580D, and uncharacterized LOC106361260, showed increased responses, while uncharacterized LOC106429057, uncharacterized BNAC06G13920D, uncharacterized LOC106452857, and uncharacterized LOC106433592 exhibited decreased responses under Gly stress. The precise biological functions of these proteins are currently unknown. However, our research observed their responses to Gly and/or Si supplementation. Further investigation is necessary to uncover their specific biological functions related to Gly-stress tolerance.

### Protein interactome analysis

The activity of a protein is often significantly influenced by its interactions with other proteins and regulatory modifications^50^. Protein-protein interactions are crucial for various biological processes, such as cell-to-cell communication and the regulation of metabolic processes^51^. The functions of the target protein can be predicted by examining its co-expression patterns and interactome analysis. Typically, the desired phenotypic traits are often produced by a series of molecular alterations caused by the interaction of genes and proteins^52^. Gaining insight into the protein interactome helps to understand the biochemical interactions, cellular signaling and signal transduction among *B. napus* leave’s diverse protein composition. Within the energy and metabolism category, some proteins, including pyruvate dehydrogenase E1 component subunit, acetyl-CoA carboxytransferase, NAD(P)H-quinone oxidoreductase subunit U, and NAD(P)H-quinone oxidoreductase subunit M are interconnected, contributing to the overall energy balance and metabolic activities within plant cells during the stress conditions. This method highlights their important contribution to plant’s metabolic processes and energy^53^.

The proteins related to photosynthesis, such as chlorophyll a-b binding protein, chlorophyll a-b binding protein 1, chlorophyll a-b binding protein CP26, photosystem I reaction center subunit II and subunit VI-1, photosystem II stability/assembly factor HCF136, and RuBisCo small subunit, are directly related with photosynthesis process and CO_2_ assimilation through the mutual interactions of that proteins.

Some proteins such as 60 S ribosomal protein L7-3, L13a-4, L18a-2-like, and 40 S ribosomal protein S3-2-like, S5-1, S16-3-like, and S7 are engaged with the process of metabolism and signal transduction. Additionally, the interactome analysis of antioxidant, sulfur assimilation, and herbicide tolerance proteins, counting L-ascorbate peroxidase, GPX 2, superoxide dismutase, and phospholipid hydroperoxide GPX 6, facilitated functional protein interactions associated with stress defense, translation, and sulfur assimilation processes in *Brassica juncea*^54^.

Determining how interrelated protein networks react to Gly toxicity reveals their possible functions and connections in plants facing Gly stress. This investigation reveals shared networks among candidate proteins involved in mitigating Gly stress in *B. napus*. The widespread use of herbicides causes oxidative stress in plants despite their weed control benefits. However, they also have negative side effects on main crops. Interactome analysis of key antioxidant defense and herbicide (Gly) tolerance proteins in *B. napus* showed that L-ascorbate peroxidase and superoxide dismutase interact with proteins involved in ascorbate and Si metabolism, enhancing the plant’s antioxidant capacity and mitigating Gly-induced oxidative stress. These enzymes play a crucial role in scavenging ROS^43^. Peroxidase degrades H_2_O_2_ to alleviate oxidative stress, while GPX utilizes peroxidase as electron acceptors to reduce oxidative damage^55^.

Glutaredoxins are oxidoreductases with electrostatic properties relevant for protein-protein interactions required for oxidoreductase activity^56^. These enzymes catalyze oxidation-reduction reactions by facilitating electron transfer between donor and acceptor molecules, participating in metabolic processes such as cellular respiration, photosynthesis, and detoxification. GRX plays a critical role in maintaining thiol-redox homeostasis and mediating redox signal transduction. Disruptions in the Grx system have been linked to the development and progression of various oxidative stress-related diseases, highlighting its importance in cellular redox balance and signaling pathways^57^. Peptide methionine sulfoxide reductase interacts with proteins that protect against oxidative stress by inactivating methionine oxidation^48^. In this current study, we found that these proteins are essential for reducing oxidative stress caused by Gly and improving Gly tolerance in *B. napus* by exogenous addition of Si.

## Conclusion

The research impact explores the proteomic insights of Si-mediated Gly-toxicity mitigation in *B. napus*. This study further highlights the urgent need for environmentally sustainable strategies to address Gly toxicity. In this study, the Si supplementation as a potential method to mitigate Gly toxicity in *B. napus* leaves provided promising results, revealing substantial alterations in protein expression profiles. Particularly the identification of key target proteins connected to antioxidant defense, herbicide tolerance and sulfur assimilation processes through interactome analyses. These findings enhance our understanding of the molecular mechanisms underlying plant responses to Gly stress and offer valuable insights into tolerance pathways. Collectively, these results lay the groundwork for further in-depth field studies to elucidate the molecular basis of Gly stress responses and explore the potential of Si in mitigating Gly toxicity in *B. napus*.

### Statistical analyses

 Statistical analysis used a two-sided t-test, with FDR (false discovery rate) correction for multiple comparisons, conducted in Perseus statistical software with default settings. Data were normalized by linear regression and transferred to Excel for detailed analysis. Peptides matching common impurities were filtered out, and at least three biological replicates were used for relative quantification and protein identification.

## Electronic supplementary material

Below is the link to the electronic supplementary material.

Supplementary Material 1

Supplementary information

Supplementary Material 1

Supplementary Material 1

Supplementary information

Supplementary information

## Data Availability

The authors declare that the main data supporting the findings of this study are available within the article and its Supplementary Information files.
