# Supplementary material for: Proteomic analysis reveals the roles of silicon in mitigating glyphosate-induced toxicity in Brassica napus L
Source: Sci Rep. 2025 Jan 20;15:2465. doi: 10.1038/s41598-025-87024-5 (PMC11743794; doi:10.1038/s41598-025-87024-5)
Supplement: Supplementary file 2 — Supplementary information [file 41598_2025_87024_MOESM2_ESM.docx]

**Suppli. Table S2.** KEGG pathways of the common identified proteins (CIPs) before filtering in *B. napus* seedlings in response to Silicon-mediated Glyphosate stress using DAVID Bioinformatics.

| Pathway ID | Pathway Name | Number of Proteins | Frequency | *p* value | Fold Enrichment |
| --- | --- | --- | --- | --- | --- |
| bna01100 | Metabolic pathways | 817 | 27.09784411 | 3.42E-23 | 1.275749372 |
| bna01110 | Biosynthesis of secondary metabolites | 419 | 13.89718076 | 1.86E-07 | 1.23939856 |
| bna03010 | Ribosome | 279 | 9.253731343 | 1.21E-54 | 2.605203712 |
| bna01200 | Carbon metabolism | 214 | 7.097844113 | 3.43E-47 | 2.82802187 |
| bna01230 | Biosynthesis of amino acids | 142 | 4.709784411 | 2.60E-18 | 2.12015012 |
| bna00195 | Photosynthesis | 96 | 3.184079602 | 1.79E-29 | 3.597223356 |
| bna00710 | Carbon fixation in photosynthetic organisms | 90 | 2.985074627 | 4.15E-35 | 4.392617807 |
| bna01240 | Biosynthesis of cofactors | 87 | 2.885572139 | 0.001263453 | 1.397779995 |
| bna00190 | Oxidative phosphorylation | 85 | 2.819237148 | 2.32E-06 | 1.679188553 |
| bna00010 | Glycolysis / Gluconeogenesis | 84 | 2.786069652 | 2.78E-15 | 2.527841543 |
| bna00630 | Glyoxylate and dicarboxylate metabolism | 80 | 2.653399668 | 1.16E-25 | 3.717130802 |
| bna00270 | Cysteine and methionine metabolism | 67 | 2.222222222 | 8.52E-08 | 1.985393524 |
| bna00620 | Pyruvate metabolism | 62 | 2.056384743 | 1.62E-10 | 2.400646976 |
| bna01210 | 2-Oxocarboxylic acid metabolism | 56 | 1.857379768 | 7.75E-07 | 2.007709538 |
| bna00520 | Amino sugar and nucleotide sugar metabolism | 55 | 1.824212272 | 0.001947595 | 1.517534101 |
| bna00020 | Citrate cycle (TCA cycle) | 54 | 1.791044776 | 1.75E-14 | 3.216747809 |
| bna01250 | Biosynthesis of nucleotide sugars | 46 | 1.525704809 | 6.20E-04 | 1.68030677 |
| bna00051 | Fructose and mannose metabolism | 44 | 1.459369818 | 1.25E-08 | 2.568369273 |
| bna00030 | Pentose phosphate pathway | 43 | 1.426202322 | 2.05E-10 | 2.955558884 |
| bna03013 | Nucleocytoplasmic transport | 43 | 1.426202322 | 0.006670916 | 1.513604398 |
| bna00260 | Glycine, serine and threonine metabolism | 40 | 1.326699834 | 1.50E-05 | 2.083593499 |
| bna00380 | Tryptophan metabolism | 37 | 1.227197347 | 0.001873644 | 1.698787545 |
| bna00480 | Glutathione metabolism | 37 | 1.227197347 | 0.043073512 | 1.377542465 |
| bna03050 | Proteasome | 35 | 1.160862355 | 5.36E-04 | 1.856443751 |
| bna00196 | Photosynthesis - antenna proteins | 33 | 1.094527363 | 1.16E-17 | 5.897370983 |
| bna00250 | Alanine, aspartate and glutamate metabolism | 32 | 1.061359867 | 1.78E-04 | 2.042379561 |
| bna00970 | Aminoacyl-tRNA biosynthesis | 31 | 1.028192371 | 0.011332855 | 1.593349763 |
| bna04146 | Peroxisome | 31 | 1.028192371 | 0.091551148 | 1.328771389 |
| bna00220 | Arginine biosynthesis | 30 | 0.995024876 | 9.00E-08 | 3.030269675 |
| bna01212 | Fatty acid metabolism | 28 | 0.928689884 | 0.025497986 | 1.534193137 |
| bna00561 | Glycerolipid metabolism | 28 | 0.928689884 | 0.058074737 | 1.420301071 |
| bna00910 | Nitrogen metabolism | 27 | 0.895522388 | 1.24E-04 | 2.256351881 |
| bna00071 | Fatty acid degradation | 26 | 0.862354892 | 9.06E-04 | 2.02695891 |
| bna00785 | Lipoic acid metabolism | 25 | 0.829187396 | 7.29E-06 | 2.765722323 |
| bna00920 | Sulfur metabolism | 23 | 0.762852405 | 0.002371564 | 1.993796839 |
| bna00860 | Porphyrin metabolism | 23 | 0.762852405 | 0.006288598 | 1.842543285 |
| bna00053 | Ascorbate and aldarate metabolism | 23 | 0.762852405 | 0.075106091 | 1.444155548 |
| bna00310 | Lysine degradation | 14 | 0.464344942 | 0.058789764 | 1.730047581 |
| bna00340 | Histidine metabolism | 12 | 0.39800995 | 0.019697274 | 2.178006329 |
| bna00300 | Lysine biosynthesis | 11 | 0.364842454 | 0.002027269 | 3.116496861 |
| bna00670 | One carbon pool by folate | 11 | 0.364842454 | 0.037834223 | 2.060909215 |
| bna00261 | Monobactam biosynthesis | 10 | 0.331674959 | 0.004150997 | 3.056850988 |
| bna00603 | Glycosphingolipid biosynthesis - globo and isoglobo series | 7 | 0.232172471 | 0.04580551 | 2.622975364 |
