# Supplementary material for: Proteomic analysis reveals the roles of silicon in mitigating glyphosate-induced toxicity in Brassica napus L
Source: Sci Rep. 2025 Jan 20;15:2465. doi: 10.1038/s41598-025-87024-5 (PMC11743794; doi:10.1038/s41598-025-87024-5)
Supplement: Supplementary file 5 — Supplementary information [file 41598_2025_87024_MOESM5_ESM.docx]

**Suppli. Table S5. Significantly enriched GO terms for the DAPs among the treatment groups in *B. napus* under Silicon-mediated Glyphosate stress**

| **GO ID** | **GO Name** | **Number of proteins** | **Frequency** | ***p*-value** | **Fold Enrichment** |
| --- | --- | --- | --- | --- | --- |
| **Biological Process** | | | | | |
| GO:0006412 | Translation | 40 | 8.097166 | 1.44E-13 | 4.124906927 |
| GO:0006457 | Protein folding | 13 | 2.631579 | 1.04E-04 | 4.032216898 |
| GO:0010498 | Proteasomal protein catabolic process | 12 | 2.42915 | 5.71E-10 | 14.49342298 |
| GO:0015979 | Photosynthesis | 10 | 2.024291 | 2.43E-04 | 4.831140992 |
| GO:0006979 | Response to oxidative stress | 9 | 1.821862 | 0.004053 | 3.551606125 |
| GO:0042026 | Protein refolding | 8 | 1.619433 | 1.51E-05 | 9.964228296 |
| GO:0009853 | Photorespiration | 7 | 1.417004 | 4.36E-06 | 15.94276527 |
| GO:0051085 | Chaperone mediated protein folding requiring cofactor | 7 | 1.417004 | 0.001247 | 5.873650364 |
| GO:0000413 | Protein peptidyl-prolyl isomerization | 6 | 1.214575 | 1.53E-04 | 11.665438 |
| GO:0015986 | ATP synthesis coupled proton transport | 6 | 1.214575 | 3.60E-04 | 9.760876698 |
| GO:0000462 | Maturation of SSU-rRNA from tricistronic rRNA transcript (SSU-rRNA, 5.8S rRNA, LSU-rRNA) | 6 | 1.214575 | 0.001517 | 7.138551615 |
| GO:0006096 | Glycolytic process | 6 | 1.214575 | 0.013408 | 4.270383555 |
| GO:0018298 | Protein-chromophore linkage | 6 | 1.214575 | 0.029923 | 3.465818538 |
| GO:0045037 | Protein import into chloroplast stroma | 5 | 1.012146 | 1.10E-05 | 33.21409432 |
| GO:0017148 | Negative regulation of translation | 5 | 1.012146 | 5.08E-04 | 13.28563773 |
| GO:0009768 | Photosynthesis, light harvesting in photosystem I | 5 | 1.012146 | 0.002589 | 8.664546344 |
| GO:0034599 | Cellular response to oxidative stress | 5 | 1.012146 | 0.00464 | 7.380909849 |
| GO:0000028 | Ribosomal small subunit assembly | 5 | 1.012146 | 0.020375 | 4.802037733 |
| GO:0009058 | Biosynthetic process | 5 | 1.012146 | 0.049486 | 3.623355744 |
| GO:0009416 | Response to light stimulus | 5 | 1.012146 | 0.077175 | 3.113821342 |
| GO:0010027 | Thylakoid membrane organization | 4 | 0.809717 | 0.001885 | 15.94276527 |
| GO:0015995 | Chlorophyll biosynthetic process | 4 | 0.809717 | 0.009429 | 9.110151585 |
| GO:0006635 | Fatty acid beta-oxidation | 4 | 0.809717 | 0.012694 | 8.175777063 |
| GO:0000470 | Maturation of LSU-rRNA | 4 | 0.809717 | 0.032998 | 5.69384474 |
| GO:0051603 | Proteolysis involved in cellular protein catabolic process | 4 | 0.809717 | 0.095684 | 3.665003511 |
| GO:0005980 | Glycogen catabolic process | 3 | 0.607287 | 0.00152 | 47.82829582 |
| GO:0030091 | Protein repair | 3 | 0.607287 | 0.00152 | 47.82829582 |
| GO:0006435 | Threonyl-tRNA aminoacylation | 3 | 0.607287 | 0.00152 | 47.82829582 |
| GO:0006526 | Arginine biosynthetic process | 3 | 0.607287 | 0.018728 | 14.06714583 |
| GO:0006086 | Acetyl-CoA biosynthetic process from pyruvate | 3 | 0.607287 | 0.038702 | 9.565659164 |
| GO:0034620 | Cellular response to unfolded protein | 3 | 0.607287 | 0.038702 | 9.565659164 |
| GO:0045036 | Protein targeting to chloroplast | 3 | 0.607287 | 0.038702 | 9.565659164 |
| GO:0019344 | Cysteine biosynthetic process | 3 | 0.607287 | 0.041588 | 9.197749196 |
| GO:0006631 | Fatty acid metabolic process | 3 | 0.607287 | 0.063847 | 7.246711488 |
| GO:0006535 | Cysteine biosynthetic process from serine | 3 | 0.607287 | 0.077987 | 6.463283219 |
| GO:0000463 | Maturation of LSU-rRNA from tricistronic rRNA transcript (SSU-rRNA, 5.8S rRNA, LSU-rRNA) | 3 | 0.607287 | 0.085391 | 6.131832797 |
| GO:0048285 | Organelle fission | 2 | 0.404858 | 0.049091 | 39.85691318 |
| GO:0006429 | Leucyl-tRNA aminoacylation | 2 | 0.404858 | 0.060983 | 31.88553055 |
| GO:0070863 | Positive regulation of protein exit from endoplasmic reticulum | 2 | 0.404858 | 0.095779 | 19.92845659 |
| GO:0016050 | Vesicle organization | 2 | 0.404858 | 0.095779 | 19.92845659 |
| GO:0061024 | Membrane organization | 2 | 0.404858 | 0.095779 | 19.92845659 |
| GO:0010124 | Phenylacetate catabolic process | 2 | 0.404858 | 0.095779 | 19.92845659 |
| **Cellular Component** | | | | | |
| GO:0005737 | Cytoplasm | 82 | 16.59919 | 4.58E-09 | 1.935088905 |
| GO:0009507 | Chloroplast | 44 | 8.906883 | 1.76E-14 | 4.037745708 |
| GO:0005829 | Cytosol | 33 | 6.680162 | 2.72E-04 | 2.002591621 |
| GO:0000786 | Nucleosome | 29 | 5.870445 | 6.37E-28 | 19.76024193 |
| GO:0005739 | Mitochondrion | 21 | 4.251012 | 0.000413 | 2.459081014 |
| GO:0022625 | Cytosolic large ribosomal subunit | 16 | 3.238866 | 1.20E-05 | 4.048478685 |
| GO:0022627 | Cytosolic small ribosomal subunit | 15 | 3.036437 | 6.51E-07 | 5.547194352 |
| GO:0009535 | Chloroplast thylakoid membrane | 13 | 2.631579 | 4.19E-05 | 4.446525749 |
| GO:0005840 | Ribosome | 13 | 2.631579 | 9.95E-05 | 4.0612672 |
| GO:0005839 | Proteasome core complex | 12 | 2.42915 | 2.93E-10 | 15.49902661 |
| GO:0046658 | Anchored component of plasma membrane | 11 | 2.226721 | 0.001762 | 3.363599119 |
| GO:0019773 | Proteasome core complex, alpha-subunit complex | 9 | 1.821862 | 5.26E-10 | 28.84541063 |
| GO:0009570 | Chloroplast stroma | 8 | 1.619433 | 0.002048 | 4.524770295 |
| GO:0005777 | Peroxisome | 7 | 1.417004 | 4.76E-02 | 2.680325766 |
| GO:0009523 | Photosystem II | 6 | 1.214575 | 1.32E-02 | 4.291052821 |
| GO:0015934 | Large ribosomal subunit | 5 | 1.012146 | 0.0015 | 10.06235254 |
| GO:0015935 | Small ribosomal subunit | 5 | 1.012146 | 0.007509 | 6.457927753 |
| GO:0009522 | Photosystem I | 5 | 1.012146 | 0.008746 | 6.18115942 |
| GO:0005759 | Mitochondrial matrix | 5 | 1.012146 | 0.035475 | 4.043749153 |
| GO:0000274 | Mitochondrial proton-transporting ATP synthase, stator stalk | 4 | 0.809717 | 1.49E-05 | 69.22898551 |
| GO:0009706 | Chloroplast inner membrane | 4 | 0.809717 | 0.015973 | 7.524889729 |
| GO:0005764 | Lysosome | 4 | 0.809717 | 0.047161 | 4.944927536 |
| GO:0009368 | Endopeptidase Clp complex | 3 | 0.607287 | 0.052337 | 8.112771739 |
| GO:0005832 | Chaperonin-containing T-complex | 3 | 0.607287 | 0.058321 | 7.635549872 |
| GO:0009526 | Plastid envelope | 3 | 0.607287 | 0.080939 | 6.331919406 |
| GO:0019774 | Proteasome core complex, beta-subunit complex | 3 | 0.607287 | 0.087827 | 6.037411527 |
| GO:0005960 | Glycine cleavage complex | 2 | 0.404858 | 0.077929 | 24.72463768 |
| **Molecular Function** | | | | | |
| GO:0003735 | Structural constituent of ribosome | 41 | 8.299595 | 6.44E-11 | 3.346292731 |
| GO:0003723 | RNA binding | 28 | 5.668016 | 1.37E-02 | 1.63409312 |
| GO:0046982 | Protein heterodimerization activity | 27 | 5.465587 | 3.86E-19 | 10.91278677 |
| GO:0016491 | Oxidoreductase activity | 16 | 3.238866 | 2.59E-02 | 1.870408125 |
| GO:0016887 | ATPase activity | 14 | 2.834008 | 0.041015 | 1.859780806 |
| GO:0003729 | mRNA binding | 14 | 2.834008 | 6.30E-02 | 1.735390899 |
| GO:0003755 | Peptidyl-prolyl cis-trans isomerase activity | 12 | 2.42915 | 1.18E-07 | 8.852659814 |
| GO:0016788 | Hydrolase activity, acting on ester bonds | 12 | 2.42915 | 5.37E-05 | 4.724476481 |
| GO:0030170 | Pyridoxal phosphate binding | 12 | 2.42915 | 8.08E-05 | 4.513980004 |
| GO:0004252 | Serine-type endopeptidase activity | 10 | 2.024291 | 3.54E-03 | 3.303710003 |
| GO:0051082 | Unfolded protein binding | 10 | 2.024291 | 0.005202 | 3.114152872 |
| GO:0019843 | rRNA binding | 9 | 1.821862 | 7.08E-05 | 6.575653564 |
| GO:0009055 | Electron carrier activity | 7 | 1.417004 | 0.034313 | 2.90654268 |
| GO:0016018 | Cyclosporin A binding | 5 | 1.012146 | 2.13E-04 | 16.51855002 |
| GO:0031072 | Heat shock protein binding | 5 | 1.012146 | 8.86E-04 | 11.5129288 |
| GO:0051537 | 2 iron, 2 sulfur cluster binding | 5 | 1.012146 | 0.015655 | 5.204474663 |
| GO:0016168 | Chlorophyll binding | 5 | 1.012146 | 0.052752 | 3.550716359 |
| GO:0070181 | Small ribosomal subunit rRNA binding | 4 | 0.809717 | 0.008401 | 9.498166259 |
| GO:0044183 | Protein binding involved in protein folding | 4 | 0.809717 | 0.023786 | 6.466836602 |
| GO:0015078 | Hydrogen ion transmembrane transporter activity | 4 | 0.809717 | 0.050054 | 4.824465401 |
| GO:0016616 | Oxidoreductase activity, acting on the CH-OH group of donors, NAD or NADP as acceptor | 4 | 0.809717 | 0.078439 | 3.999227899 |
| GO:0008887 | Glycerate kinase activity | 3 | 0.607287 | 0.001014 | 56.98899756 |
| GO:0004829 | Threonine-tRNA ligase activity | 3 | 0.607287 | 0.001675 | 45.59119804 |
| GO:0008184 | Glycogen phosphorylase activity | 3 | 0.607287 | 0.001675 | 45.59119804 |
| GO:0033743 | Peptide-methionine (R)-S-oxide reductase activity | 3 | 0.607287 | 0.001675 | 45.59119804 |
| GO:0048027 | mRNA 5'-UTR binding | 3 | 0.607287 | 0.004569 | 28.49449878 |
| GO:0004615 | Phosphomannomutase activity | 3 | 0.607287 | 0.005823 | 25.32844336 |
| GO:0047958 | Glycine:2-oxoglutarate aminotransferase activity | 3 | 0.607287 | 0.007216 | 22.79559902 |
| GO:0008453 | Alanine-glyoxylate transaminase activity | 3 | 0.607287 | 0.008743 | 20.72327184 |
| GO:0016149 | Translation release factor activity, codon specific | 3 | 0.607287 | 0.012187 | 17.53507617 |
| GO:0004021 | L-alanine:2-oxoglutarate aminotransferase activity | 3 | 0.607287 | 0.016124 | 15.19706601 |
| GO:0004602 | Glutathione peroxidase activity | 3 | 0.607287 | 0.027949 | 11.39779951 |
| GO:0004124 | Cysteine synthase activity | 3 | 0.607287 | 0.030627 | 10.85504715 |
| GO:0004427 | Inorganic diphosphatase activity | 3 | 0.607287 | 0.039233 | 9.498166259 |
| GO:0004739 | Pyruvate dehydrogenase (acetyl-transferring) activity | 3 | 0.607287 | 0.042282 | 9.118239609 |
| GO:0051787 | Misfolded protein binding | 3 | 0.607287 | 0.065869 | 7.123624694 |
| GO:0004298 | Threonine-type endopeptidase activity | 3 | 0.607287 | 0.080886 | 6.332110839 |
| GO:0008863 | Formate dehydrogenase (NAD+) activity | 2 | 0.404858 | 0.038871 | 50.65688672 |
| GO:0004823 | Leucine-tRNA ligase activity | 2 | 0.404858 | 0.063944 | 30.39413203 |
| GO:0004742 | Dihydrolipoyllysine-residue acetyltransferase activity | 2 | 0.404858 | 0.076235 | 25.32844336 |
| GO:0031956 | Medium-chain fatty acid-CoA ligase activity | 2 | 0.404858 | 0.088365 | 21.71009431 |
| GO:0010277 | Chlorophyllide a oxygenase [overall] activity | 2 | 0.404858 | 0.088365 | 21.71009431 |
| GO:0016630 | Protochlorophyllide reductase activity | 2 | 0.404858 | 0.088365 | 21.71009431 |
