# Supplementary material for: Proteomic analysis reveals the roles of silicon in mitigating glyphosate-induced toxicity in Brassica napus L
Source: Sci Rep. 2025 Jan 20;15:2465. doi: 10.1038/s41598-025-87024-5 (PMC11743794; doi:10.1038/s41598-025-87024-5)
Supplement: Supplementary file 6 — Supplementary information [file 41598_2025_87024_MOESM6_ESM.docx]

**Suppli. Table S6.** KEGG pathway analysis of differentially abundant proteins (DAPs) after filtering in leaves of *B. napus* seedlings exposed to Silicon-mediated Glyphosate stress for 7 days

| Pathway ID | Pathway Name | Number of Proteins | Frequency | *p* value | Fold Enrichment |
| --- | --- | --- | --- | --- | --- |
| bna01100 | Metabolic pathways | 69 | 13.96761134 | 3.04E-02 | 1.199905233 |
| bna01110 | Biosynthesis of secondary metabolites | 41 | 8.299595142 | 3.52E-02 | 1.327126041 |
| bna03010 | Ribosome | 23 | 4.655870445 | 2.37E-04 | 2.346894936 |
| bna01200 | Carbon metabolism | 17 | 3.441295547 | 1.16E-03 | 2.491728672 |
| bna00630 | Glyoxylate and dicarboxylate metabolism | 11 | 2.226720648 | 2.31933E-05 | 5.669037338 |
| bna01230 | Biosynthesis of amino acids | 11 | 2.226720648 | 8.51E-02 | 1.788053928 |
| bna03050 | Proteasome | 8 | 1.619433198 | 1.47E-03 | 4.733741616 |
| bna00270 | Cysteine and methionine metabolism | 8 | 1.619433198 | 0.034513358 | 2.585772154 |
| bna00250 | Alanine, aspartate and glutamate metabolism | 6 | 1.214574899 | 1.33E-02 | 4.246885279 |
| bna01212 | Fatty acid metabolism | 6 | 1.214574899 | 2.30E-02 | 3.686856451 |
| bna00260 | Glycine, serine and threonine metabolism | 6 | 1.214574899 | 0.028741367 | 3.476724736 |
| bna04146 | Peroxisome | 6 | 1.214574899 | 0.066195445 | 2.750032271 |
| bna00220 | Arginine biosynthesis | 5 | 1.012145749 | 0.01286991 | 5.482090474 |
| bna00071 | Fatty acid degradation | 5 | 1.012145749 | 2.85E-02 | 4.301332526 |
| bna01040 | Biosynthesis of unsaturated fatty acids | 4 | 0.809716599 | 1.34E-02 | 7.988188976 |
| bna00196 | Photosynthesis - antenna proteins | 4 | 0.809716599 | 0.014755985 | 7.712734184 |
| bna00592 | Alpha-Linolenic acid metabolism | 4 | 0.809716599 | 0.092687659 | 3.69701308 |
